# CDK9: A Comprehensive Review of Its Biology, and Its Role as a Potential Target for Anti-Cancer Agents

**DOI:** 10.3389/fonc.2021.678559

**Published:** 2021-05-10

**Authors:** Abel Tesfaye Anshabo, Robert Milne, Shudong Wang, Hugo Albrecht

**Affiliations:** Drug Discovery and Development, Centre for Cancer Diagnostics and Therapeutics, Clinical and Health Sciences, University of South Australia, Adelaide, SA, Australia

**Keywords:** cancer, CDKs, transcription, P-TEFb, CDK9 inhibitors

## Abstract

Cyclin-dependent kinases (CDKs) are proteins pivotal to a wide range of cellular functions, most importantly cell division and transcription, and their dysregulations have been implicated as prominent drivers of tumorigenesis. Besides the well-established role of cell cycle CDKs in cancer, the involvement of transcriptional CDKs has been confirmed more recently. Most cancers overtly employ CDKs that serve as key regulators of transcription (e.g., CDK9) for a continuous production of short-lived gene products that maintain their survival. As such, dysregulation of the CDK9 pathway has been observed in various hematological and solid malignancies, making it a valuable anticancer target. This therapeutic potential has been utilized for the discovery of CDK9 inhibitors, some of which have entered human clinical trials. This review provides a comprehensive discussion on the structure and biology of CDK9, its role in solid and hematological cancers, and an updated review of the available inhibitors currently being investigated in preclinical and clinical settings.

## Introduction

Protein kinases are a large family of enzymes that regulate most eukaryotic cellular processes and signaling pathways through protein phosphorylation. This can activate or inhibit enzymes, increase protein-protein interactions, change cellular localization, or generate a site for the recruitment of proteins (1, 2). Protein kinases are themselves regulated by different transcriptional and post-translational modifications. The overall outcome is the regulation of cellular proliferation, apoptosis, and differentiation (1, 2). The human genome encodes more than 500 protein kinase genes (3). Due to their key functions, deregulation of protein kinase activity as a consequence of genetic mutation or the absence of a negative regulator is associated with a number of pathological disorders (e.g., cancer and inflammatory disorders) (1, 2).

Cyclin-dependent kinases (CDKs) are serine (Ser)/threonine (Thr) protein kinases activated by regulatory cyclin proteins (4). The human genome encodes twenty CDKs (numbered 1-20) belonging to the CDK- and CDK-like branch of the CMGC subfamily of human kinases which include the **c**yclin-dependent, **m**itogen-activated, **g**lycogen synthase and **C**DC-like kinases (4). CDKs together with their cyclin partners play specific roles in numerous cellular processes, such as cell division and transcription, in response to intra- and extra-cellular signals (5). CDK proteins have a two-lobed structure with the active site sandwiched between an amino (N)-terminal lobe comprised mostly of β-sheets and a carboxyl (C)-terminal lobe of α-helices. CDKs are regulated by binding of the cyclin subunits (themselves regulated by formation and degradation) and phosphorylation of conserved residues in the T- and glycine (G) - rich loop structures within the CDK (4). Based on their evolutionary relationships and main functional roles, CDKs may be divided into two main groups: those which regulate the cell cycle (e.g. CDKs 1-7, 14-18) and those regulating transcription (e.g. CDKs 7-13, 18-20) (5). Owing to these central regulatory functions it is perhaps not surprising that dysregulation of CDK activity is closely associated with human malignancies. This is particularly so for CDK9, a key regulator of transcription which is overtly employed by cancer cells for the constant production of short-lived proteins that maintains their survival. In this review, the role of CDK9 in the pathogenesis of hematological and solid cancers will be discussed together with a comprehensive review of its discovery, structure, biological function, regulation, and the available pharmacological inhibitors currently being investigated as anticancer agents.

## Discovery of P-TEFb (CDK9-Cyclin T)

Identification of the regulation of gene transcription at the elongation phase began with the observation that cells treated with the nucleoside analogue 5,6-dichloro-1-β-D-ribofuranosyl benzimidazole (DRB), a general inhibitor of transcription, continued to produce short capped ribonucleic acids (RNA) (6). Initially, it was proposed that DRB might cause premature transcription termination (6). Later evidence, however, indicated that it might act at the elongation step as it lacks an inhibitory effect on the transcription efficacy of elongating RNA polymerase II (RNAP II) that has already synthesized RNAs past the transcription elongation checkpoint (7, 8). Parallel to these observations, there was growing evidence that RNAP II pauses transcription shortly after its initiation (9). This was shown by the appearance of long RNA transcripts from a deoxyribonucleic acid (DNA) template in the presence of nuclear extracts at a rate much slower than the appearance of short-length RNA (9). Furthermore, purified RNAP II elongation complexes (cleared of other transcription factors) were only capable of producing short RNAs as compared to incubation of RNAP II with nuclear extracts, indicating the presence of stimulatory transcription elongation factors (9).

Simultaneously, certain agents (e.g., high salt treatment) were shown to initially reduce the elongation rate, but with time caused an increase in the number of long transcripts (8, 9). The two opposite effects were ascribed to dual inhibition of elongation factors and disruption of a transcription pause (9). These findings led to a model suggesting the presence of a block-relief elongation control system directly affected by DRB. The opposing proteins responsible for such block-relief control were subsequently named Positive Transcription Elongation Factor (P-TEF, the one that relieves the pause and is affected by DRB) and Negative Transcription Elongation Factor (N-TEF, the protein preventing transcription elongation) (9).

Following the identification of these two factors, further characterization of the P-TEF complex revealed a component named P-TEFb as the primary target of DRB during transcription elongation (10). The removal of P-TEFb from a nuclear extract incubated with a DNA template totally abolished the production of long RNA transcripts with no effect seen on the level of short-length RNAs (10). The actual mechanism through which P-TEFb relieves the transcriptional pause was not clear until discovery of the importance of phosphorylation of the carboxyl terminal domain (CTD) on the largest subunit (Rpb1) of RNAP II for productive transcription elongation. Notably, hypo-and hyper-phosphorylated states paralleled a pause and productive elongation, respectively (11), and this was accompanied with decreasing long RNA production when RNAP II CTD became increasingly truncated (12). Coincidentally, P-TEFb was shown to have kinase activity specifically directed against the CTD (12).

Prior to the identification of P-TEFb a protein originally named PITALRE (for its characteristic **P**roline, **I**soleucine, **T**hreonine, **A**lanine, **L**eucine, **A**rginine, **G**lutamic acid motif) has been cloned during efforts to discover Ser/Thr protein kinases with crucial functions during the cell cycle. Its major characteristics were a wide tissue distribution, close similarity with cell cycle kinases, and localization within the nucleus (13). Its real identity and potential role in transcription, however, was later discovered during a search for subunits of P-TEFb. The sequence and function of PITALRE were shown to be identical to that of the small subunit of P-TEFb (14). Like P-TEFb, PITALRE phosphorylated RNAP II CTD and its removal led to inhibition of long RNA production in a DRB-sensitive manner (14). This led to the identification of PITALRE as the kinase component of P-TEFb. Subsequently, the large subunit of P-TEFb was shown to have structural signatures of cyclin proteins (*i.e.*, two sequences of five α-helices) and was required for phosphorylation of the CTD and productive elongation (15).

**Figure 1:**
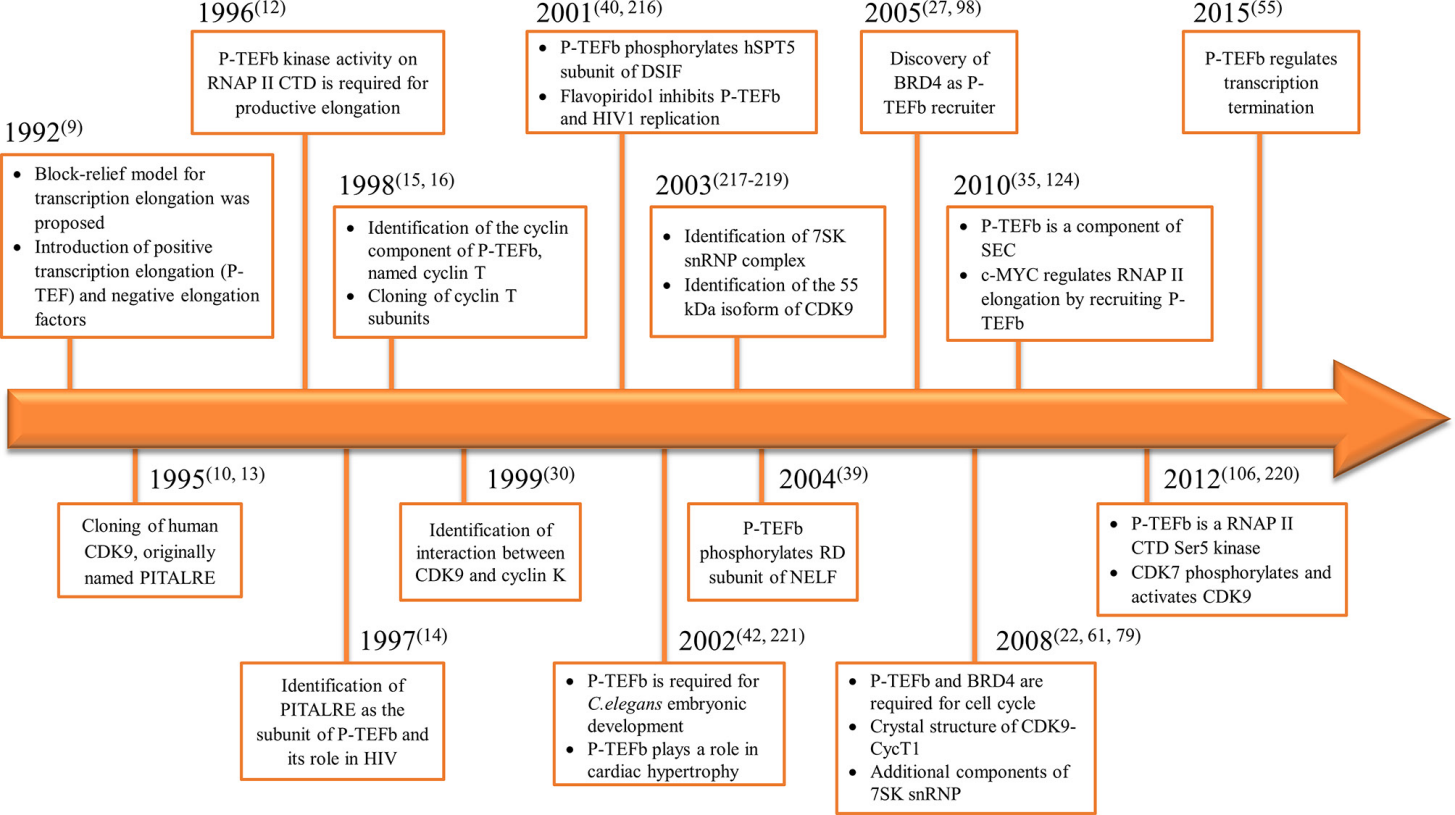
Timeline for the discovery of P-TEFb and its biological roles.

Consequently, the kinase subunit was renamed CDK9 and its cyclin partner cyclin T (with three subunits identified cyclin T1, T2a, and T2b) (15, 16). Simultaneously, a protein derived from the human immunodeficiency virus 1 (HIV1), known as Trans-Activator of Transcription (TAT), was shown to have a role in viral transcription through the formation of a complex which contained P-TEFb (17). We summarized the timeline for P-TEFb biology key findings in **Figure 1**.

## Structure of the CDK9-Cyclin T Complex

The eukaryotic kinases consist of conserved sequences of 250 to 300 amino acids that form a common catalytical core called the kinase domain (18). The kinase domain contains twelve conserved sub-domains that fold into a bilobal tertiary structure with smaller N-terminal and larger C-terminal lobes. The N-terminal region contains mainly β strands while α-helices form a major part of the C-terminal region (19–21).

### Structure of CDK9

**Figure 2:**
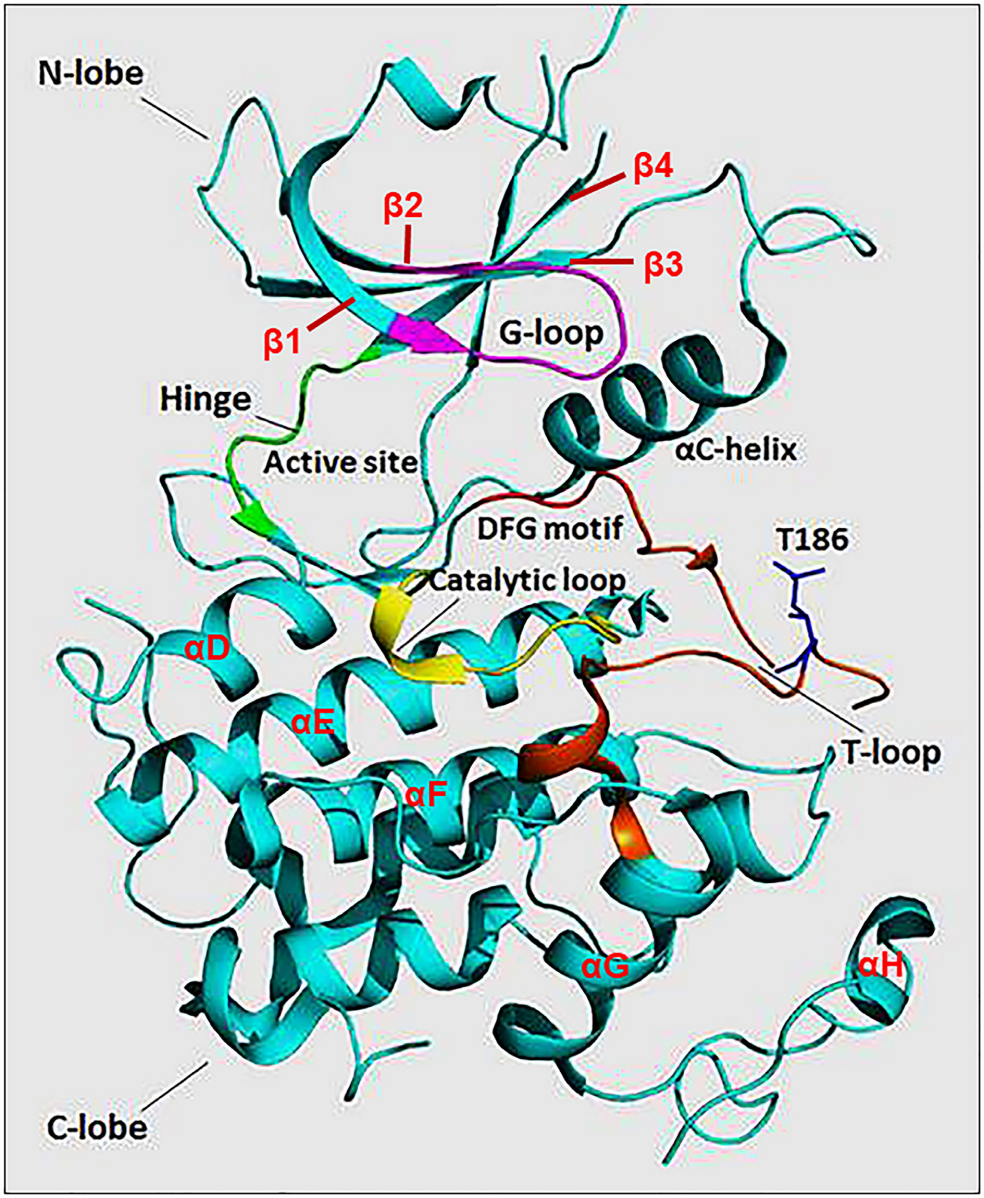
The protein structure of monomeric CDK9 (Protein Data Bank: 3BLQ). The bilobal CDK9 structure is dominated by N-terminal β-sheets (1-4 are shown) and C-terminal α-helices (D-H are shown). The C-terminal also contains β-sheets (6-9, not shown). The two lobes are connected by a hinge region (green) that binds the adenine moiety of adenosine triphosphate (ATP). The N-terminus contains an αC helix and glycine-rich loop (G-loop, purple) which binds cyclin and ATP, respectively. The C-terminus comprises the catalytic loop (yellow), T-loop (brown), and DFG motif that binds Mg^+2^. The threonine residue (Thr186) involved in CDK9 activation is found in the T-loop structure.

The N-terminal lobe of CDK9, which spans from amino acid residue 16 to 108, comprises five β structures (β1-5) and one α helix (αC) (**Figure 2**) (22). The C-terminal lobe (residues 109-330) is composed of seven α-helices (αD-J) and four β strands (β6-9) (22). The interaction between CDK9 and cyclin T1 occurs mainly through the αC helix located on the N-terminal lobe (**Figures 2** and **4**). This helix contains a peptide sequence highly conserved across CDKs (PITALRE in CDK9) and serves as a site for interaction with cyclin during the activation of CDKs (22).

**Figure 3:**
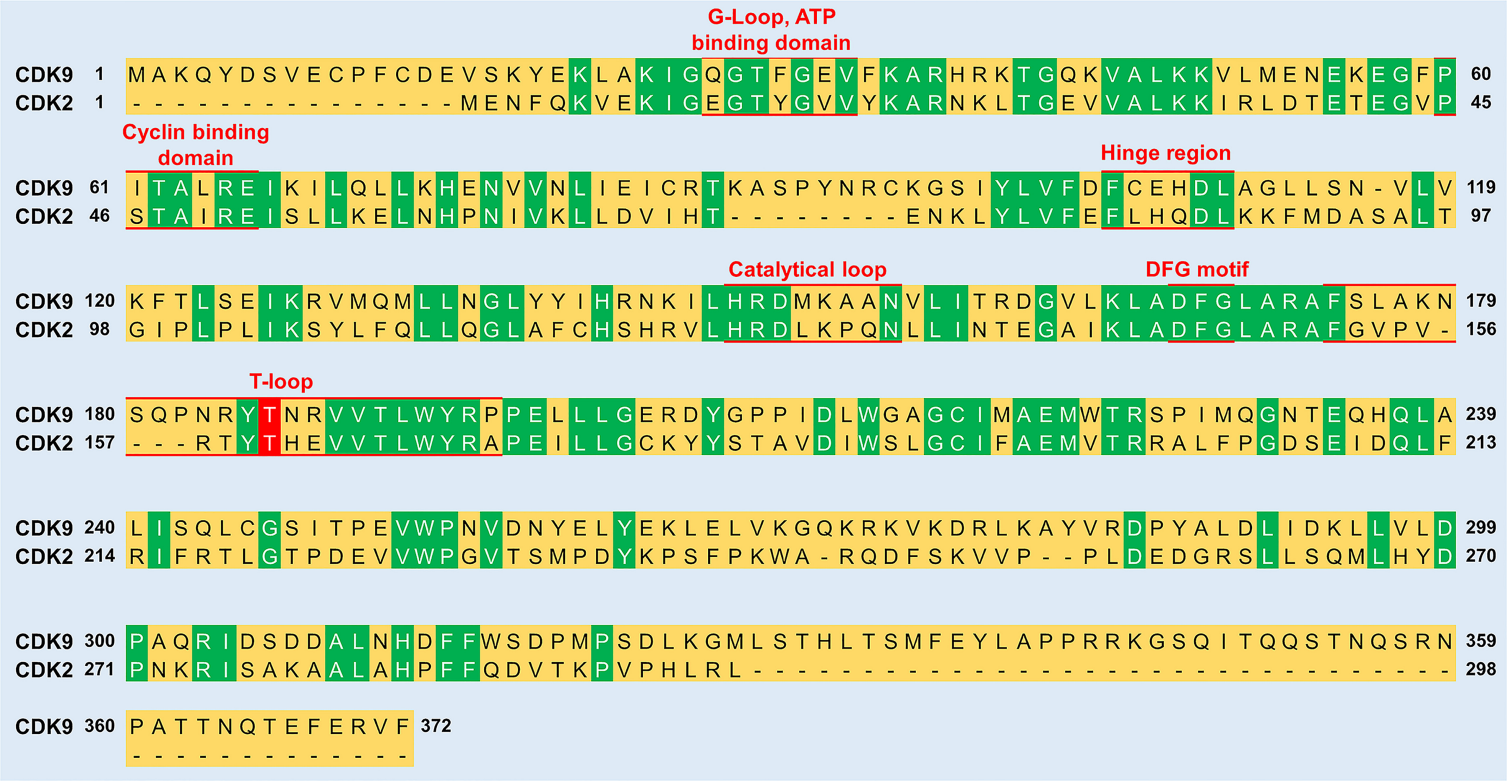
Sequence comparison between CDK9 and CDK2. The sequence identity between the two proteins is 31.9%. Green color indicates residues conserved between CDK9 and CDK2. Red underlined residues indicate the different functional subunits of the kinases. In the T-loop, the phosphorylation of a conserved threonine residue (labelled red) is vital for the activation of both CDK9 (Thr186) and CDK2 (Thr160). The sequence alignment was generated and % sequence similarity determined using UniProt (https://www.uniprot.org/align/) and sequence identifiers were P50750 for CDK9 and P24941 for CDK2.

The ***Adenosine Triphosphate* (*ATP) Binding Motif*** forms a cleft between the N- and C-terminal lobes and is highly conserved among CDKs (**Figures 2** and **3**) (22). In this site, the adenine moiety of ATP is inserted deep into the cleft and the phosphate groups are positioned toward the exterior (18). The hydrophobic pocket harboring the adenine moiety is located between the β-sheets of the N lobe and a hinge region loop which connects the two lobes (20, 22). In this region, the ATP adenine nitrogen atoms, N6 and N1, form hydrogen bonds with the main chain oxygen and nitrogen of Asp104 and Cys106 residues, respectively (22). In addition to hydrogen bonds, multiple interactions of the purine ring with aliphatic and aromatic residues of the hinge region also help in anchoring the adenine moiety (22). The α and β non-transferable phosphates of ATP are held in position through ionic and hydrogen bonds with residues located in the G-loop between β1 and β2 (**Figure 2**) (20, 22). The β- and γ-phosphates in concert with an aspartate residue and two water molecules form coordination bonds with a cationic Mg^+2^ cofactor. The aspartate residue involved in this process (Asp167 in CDK9, Asp145 in CDK2) belongs to a ‘DFG’ motif located in a loop between β8 and β9 (**Figure 2**) (18, 20, 22).

The ***Substrate Recognition Motif*** is located in the cleft between the N- and C- lobes in close proximity to the γ-phosphate of ATP (20). In general, CDKs have a strong preference for substrate motifs which have a proline residue immediately flanking a phospho-Ser or phospho-Thr residue (*i.e.* **Ser/Thr**-Pro-X-Arg/Lys) (4). This is ascribed to the presence of a hydrophobic pocket (created by the interaction between Val190 and Arg195 of CDK9) that can only accommodate a proline residue at the +1 position relative to the phosphorylation site (22). While the recognition motif is universal for the CDK family, subtle differences exist between members in their substrate preference, depending on the stringent requirement of a specific residue at the +3 position (4). This has been exemplified with the substrate recognition differences between CDK2 and CDK9, where the latter displayed a strict requirement for the Ser/Thr-Pro-X-**Arg/Lys** consensus (23).

The residues of the ***Catalytic Loop*** are highly conserved among protein kinases suggesting a similar catalytic mechanism (**Figures 2** and **3**) (24). The main mechanism involves transformation of the hydroxyl group of the Ser or Thr residue on the substrate into a nucleophile capable of attacking the γ-phosphate of ATP (24). A conserved aspartate (Asp149 in CDK9) facilitates this by acting as a general base that helps align the substrate oxygen (22, 24). Two additional residues, namely Lys151 and Thr165, have been suggested to play a secondary role by orientating the substrate (22).

***T-Loop***: When cyclin is not bound, the catalytic cleft is completely blocked by a C-terminal loop named the T-loop or activation segment (**Figures 2** and **3**) (20). This conformation hinders critical interactions between different residues and the non-transferable phosphates of ATP vital for locking ATP in a catalytically favorable position. During activation, binding of cyclin physically pulls the T-loop outward from the catalytic cleft and exposes a threonine residue found in the loop (Thr186 in CDK9 and Thr160 in CDK2; **Figure 3**) (4, 20, 22). The phosphorylation of this residue stabilizes the T-loop in an open position, as phospho-Thr186 coordinates the formation of an intramolecular hydrogen bonding network containing Arg148 and Arg172, resulting in a fully active kinase protein (22).

### Structure of Cyclins T and K

In general, cyclins are characterized by the presence of two similar regions, each having five α-helices and a short ending helix (N- or C- terminal helices). Each stalk of helices forms a separate canonical cyclin box, generally composed of approximately 100 conserved amino acid residues each. The two regions are arranged around central helices in an antiparallel fashion, forming a rigid structure that is liable to minor conformational change during binding to a kinase protein (22). Although both the N- and C- terminal regions of cyclins make contact with the kinase protein, interactions leading to activation of the kinase occur mainly through the N-terminal cyclin box region (4, 19). Despite their similarity in structure and sequence, major differences in regions outside of the cyclin box between those cyclins involved in cell cycle control (e.g., cyclins A, B, E) and those in transcription (e.g. cyclins T, H, C, K) are observed. Notably, there is a clear variation in the length and orientation of the short-ending helices (22).

The major cyclin partner of CDK9, cyclin T (‘T’ named after the first letter of HIV TAT), has a close similarity to cyclin C and cyclin H (15, 17). Three cyclin T members are known, namely T1 (726 residues), T2a (663 residues), and T2b (730 residues), which have a high degree of identity (81%) in their cyclin box region (16). HIV TAT interacts only with cyclin T1 in complex with CDK9 to mediate HIV transcription (25, 26). Human cyclin T1 (**Figure 7B**) contains a recognition motif for TAT-Transactivation Response Element (TAR) complex (TRM, residues 254-272) (26) found downstream of the N-terminal cyclin box (residues 1-263), a putative coiled-coil motif (residues 379–430), histidine-rich motif (residues 506–530) (27, 28), and a C-terminal PEST (Pro-Glu-Ser-Thr) sequence (residues 709–726) (29). CDK9 also forms a complex with cyclin K, which displays CTD kinase activity, despite the fact that cyclin K only shares 29% identity with cyclin T at the amino acid level (30). Nonetheless, the CDK9 interaction site is conserved among cyclin T and K, explaining their similar modes of interaction (31).

### Interactions Between CDK9 and Cyclin T1

**Figure 4:**
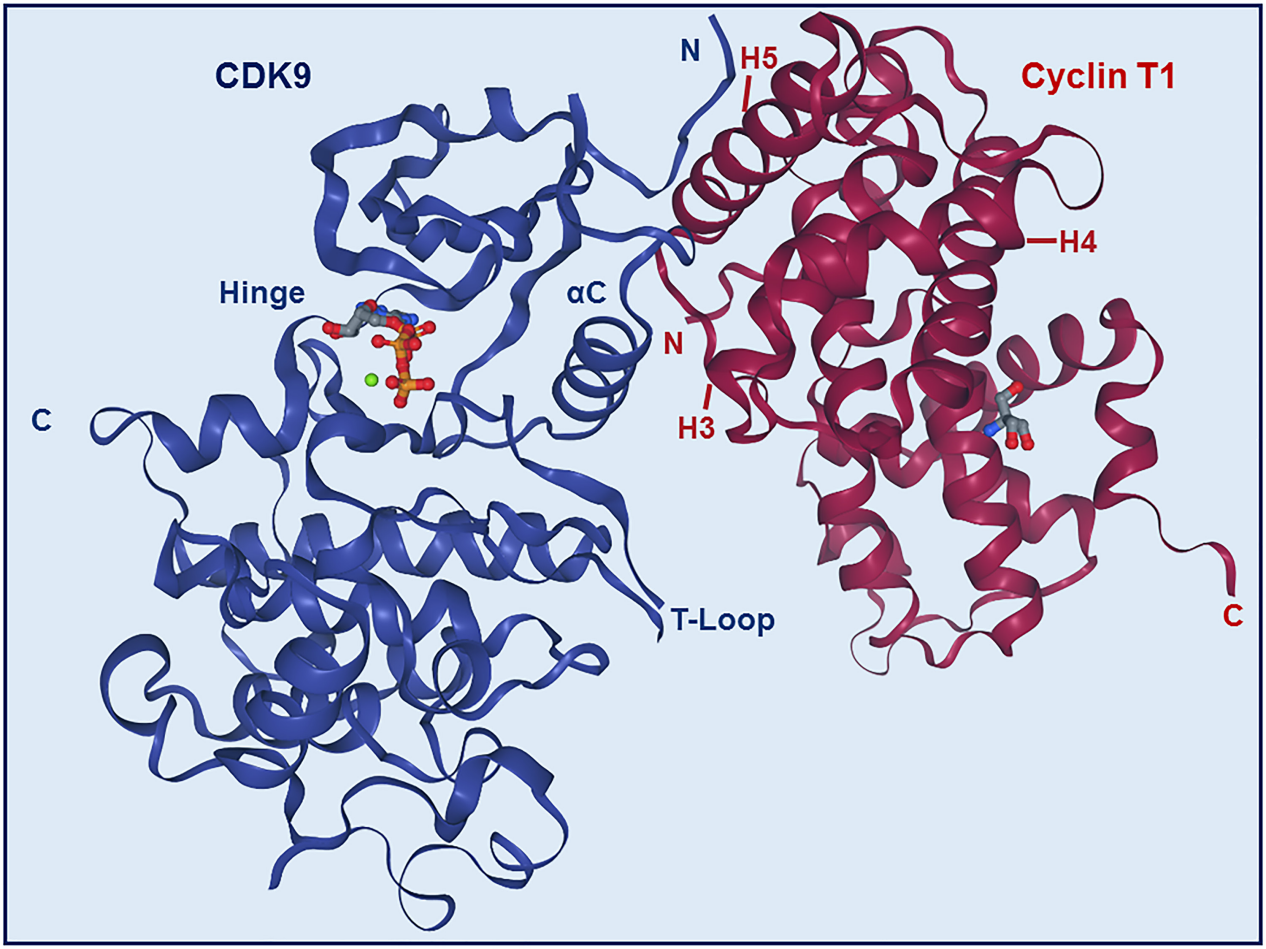
Structure of CDK9-Cyclin T1 complex. Stereo ribbon plot of the 3D structure of CDK9-cyclin T1 in complex with ATP (Protein data bank: 3BLQ). CDK9 (blue) and cyclin T1 (dark red) make contact through the αC helix and β4 strand of CDK9 and H3, H4, and H5 helices of cyclin T1. The CDK9 T-loop and hinge regions are indicated.

Most of the binding between CDK9 and cyclin T1 involves interactions between the H3, H4, and H5 helices of the cyclin and the αC helix and β4 strand of the CDK (**Figures 2** and **4**) (22). The H5 helix interacts with the αC helix and enforces an active conformation. While this mechanism is common across CDKs, in contrast to most other CDKs, the N-terminal short helix (H_N_) in cyclin T1 makes no contact with CDK9, which gives forth to a more solvent-exposed kinase surface.

## Biological Functions of P-TEFb

### Control of Transcriptional Elongation and Termination

Normal cellular growth and development are dependent on efficient and intricate regulation of gene expression. This regulation primarily occurs during transcription, which is the initial step of expression and revolves around the interaction of multiple host factors with the CTD of RNAP II. The CTD consists of tandem heptapeptide repeats (52 in mammals) of the consensus sequence Tyr-Ser-Pro-Thr-Ser-Pro-Ser (Y_1_S_2_P_3_T_4_S_5_P_6_S_7_) (32, 33). These host factors guide RNAP II to gain access to transcription sites, initiate and elongate transcription, and couple transcription of messenger RNA (mRNA) with its processing, including capping, splicing, and polyadenylation (32–34).

Formerly, transcriptional initiation was viewed as the main checkpoint for regulating transcription, while little emphasis was given to transcriptional elongation. This viewpoint, however, has changed considerably as it now becomes apparent that elongation is a highly dynamic and strictly regulated stage of transcription (32). Shortly after RNAP II initiates transcription and synthesizes 20-50 nucleotides of the nascent RNA, it is engaged by factors which significantly hinder its ability to continue elongation (9, 33). In fact, the majority of RNAP II is found paused at the promoter-proximal regions of most mammalian genes, ready to resume transcription elongation (35). While the exact purpose of RNAP II pausing is not clarified, some of the proposed functions include (1): increasing the accessibility of genes that would otherwise have a high chance of being condensed into nucleosomes (2), allowing rapid and synchronous gene activation (3), integrating multiple regulatory signals, and (4) a checkpoint for coupling elongation with 5’ end-capping of nascent RNA (36). Two factors, namely DRB sensitivity-inducing factor (DSIF) and negative elongation factor (NELF), cooperate in pausing RNAP II (37, 38).

**Figure 5:**
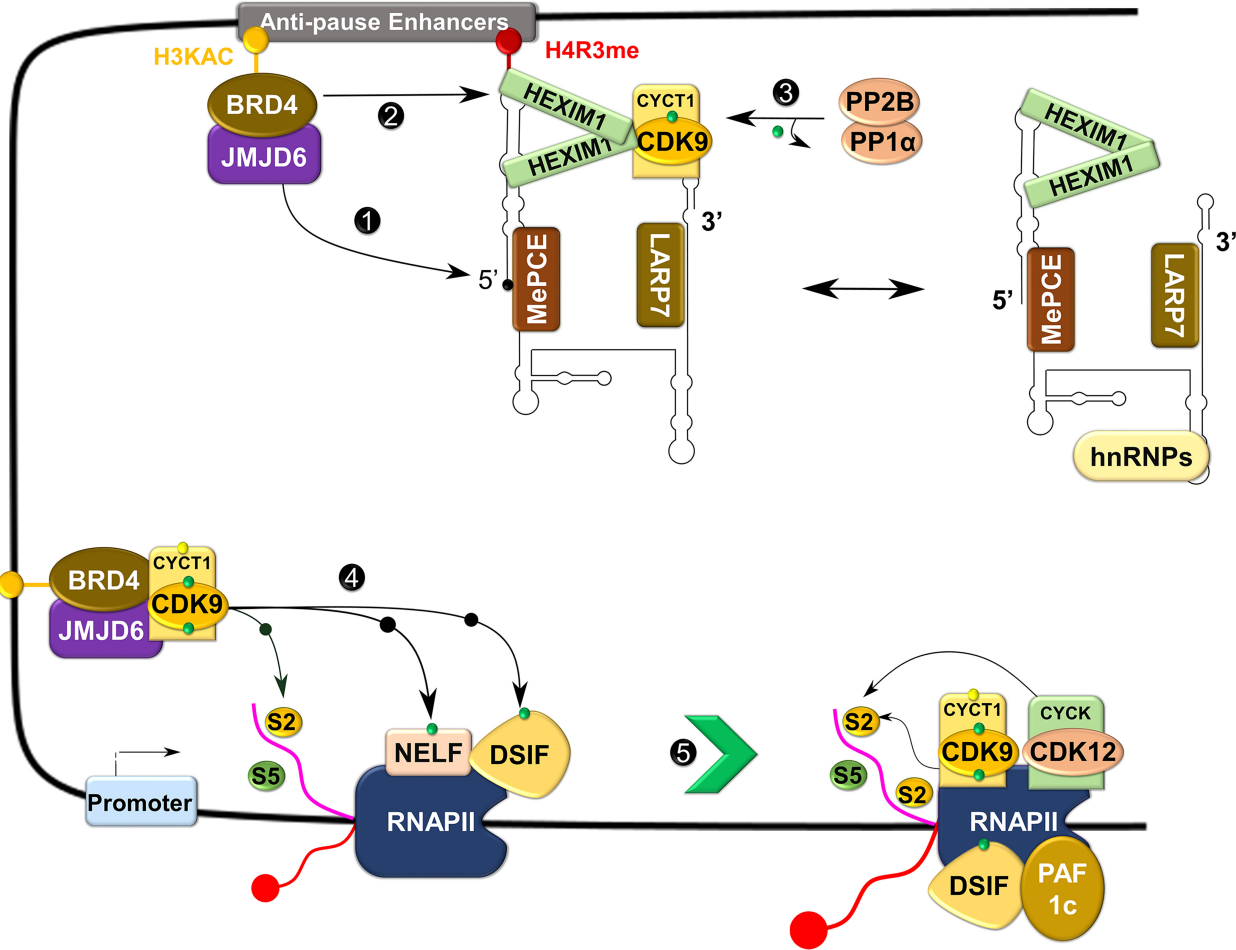
Control of transcriptional elongation by P-TEFb. During active transcription, BRD4 recruits JMJD6 to 7SK snRNP anchored to anti-pause enhancers on chromatin. JMJD6 demethylates both H4R3me and the 5’ hairpin of 7SK RNA, breaking chromatin binding of the former and exposing the latter for degradation (Labelled as 1). Concurrently, acetylated histone (H3KAC)-bound BRD4 interacts with and extracts P-TEFb from 7SK snRNP (2). Protein phosphatases (PP2B and PP1α) also assist in the release of P-TEFb from 7SK snRNP by dephosphorylating CDK9 pThr186 (3). After release, CDK9 is re-phosphorylated on Thr186 by CDK7 and delivered by BRD4-JMJD6 to RNAP II that has been paused in the proximal promoter region. At this site, P-TEFb phosphorylates DSIF, NELF, and RNAP II CTD (4), allowing productive elongation (5).

The RNAP II requires the kinase activity of P-TEFb to overcome the pause and continue elongation (**Figure 5**) (14). Upon recruitment to the paused site by bromodomain-containing protein 4 (BRD4), P-TEFb phosphorylates one of the four subunits of NELF (NELF-E or RD) and the human SPT5 (hSPT5) subunit of DSIF (39, 40). These phosphorylation steps liberate NELF from RNAP II, while converting DSIF into a positive elongation factor to track along elongating RNAP II (39, 41). Simultaneously, P-TEFb also phosphorylates the CTD of RNAP II, primarily on the Ser2 residue (**Figure 5**) (42–44). Phosphorylation of the CTD is vital for the efficient coupling of transcription elongation and pre-mRNA processing (45, 46). Ser2 phosphorylated CTD recruits chromatin-modifying factors, elongation factors, the co-transcriptional splicing machinery, and pre-mRNA 3’ end processing factors (47–50). In addition to P-TEFb, other CTD kinases such as CDK12, CDK13, and BRD4 (an atypical kinase) have been shown to phosphorylate the CTD on the Ser2 residue, although the direct role of this phosphorylation by these kinases, particularly CDKs 12 and 13, remains elusive (51–53).

In addition to the well-known role of P-TEFb in transcriptional elongation, an interdependence between its role in releasing RNAP II from its pause and transcriptional initiation has also been identified (54, 55). Namely, a high degree of RNAP II promoter-proximal pausing, induced by CRISPR-Cas9-based or small-molecule inhibition of CDK9, limits the frequency of any new round of transcriptional initiation (referred to as ‘pause-initiation’ limit), giving an insight into how cells maintain the appropriate quantity of RNA from particular genes (54, 55).

P-TEFb also plays a direct regulatory role in terminating transcription. Analogous to promoter-proximal pausing, an additional major elongation checkpoint that is dependent on P-TEFb has been identified near the terminal poly(A) sites (56). Most RNAP II that escapes promoter-proximal pausing despite the presence of CDK9 inhibitors (e.g., KM05382 and DRB) has the capacity to elongate transcription, but prematurely terminate transcription near poly(A) sites. This termination was directly linked with a loss of association of P-TEFb, DSIF, and poly(A) factors (e.g., Ssu72 and CstF64) to RNAP II (56). Beyond the poly(A) site, CDK9 regulates transcriptional termination by phosphorylating Xrn2, a 5’-3’exoribonuclease, on Thr439 and enhances its cleavage of the RNA transcript from RNAP II (57). Furthermore, CDK9 phosphorylates and inhibits protein phosphatase 1 (PP1) activity on DSIF and RNAP II until the complex reaches transcription termination sites. At these sites, PP1 becomes activated and dephosphorylates DSIF, which leads to the termination of transcription (58).

### P-TEFb in the Cell Cycle

Timely progression through each phase of the cell cycle is controlled by spatio-temporal expression of different cyclins (e.g. cyclins D, E, A, and B) which control the kinase activity of their respective cell cycle CDKs (5). Unlike cell cycle CDKs, the expression of CDK9 and its cyclin partner, as well as its kinase activity, does not change in a cell cycle-dependent manner (13, 59). This observation has led the conventional view that P-TEFb is only a transcriptional CDK with a limited role in the cell cycle. Contrary to this view, silencing of *Cdk9* by RNA interference (RNAi) induced the arrest of *Drosophila* cells in the G_1_ stage of their cycle (60). The missing mechanistic link was provided by BRD4, a mitotic bookmark that remains attached to chromatin during mitosis when all other transcription factors have dissociated (61–64). This “bookmarking” is vital for prompt re-activation of transcription after mitosis (61, 63). Beginning around mid to late anaphase, BRD4 marks many M/G_1_ genes and in concert with jumonji C-domain-containing protein 6 (JMJD6) induces promoter-proximal pause release, and recruits P-TEFb for RNAPII, NELF and DSIF phosphorylation (**Figure 5**). Subsequently, this results in the expression of key G_1_ genes to promote the progression of cells into their S phase (62, 63). Abrogation of this process through BRD4 knockdown reduces the binding of P-TEFb to mitotic chromosomes and the expression of key G_1_ and G_1_-associated genes, leading to cell cycle arrest and apoptosis (62).

### P-TEFb in Cellular Differentiation

P-TEFb influences many cellular differentiation programs (65–70). For example, CDK9-cyclin T2a interacts directly with myoblast determination protein 1 (MyoD), a basic helix-loop-helix muscle differentiation factor, and promotes MyoD-dependent transcription and activation of myogenic differentiation (66). Similarly, CDK9-cyclin T1 activates muscle differentiation programs by stimulating the transcription program of myocyte enhancer factor 2 [MEF2 (67)], indicating interaction with MyoD or MEFs is dictated by the particular cyclin T.

P-TEFb is also required for the differentiation of monocytes (70), lymphocytes (68), adipocytes (71), and neurons (69, 72). Treatment of monocytes with a potent inducer of differentiation, phorbol 12-myristate 13-acetate, induces increased expression of cyclin T1 and of P-TEFb activity (70). Similarly, the expression of both CDK9 and cyclin T1 is linked to a particular stage of lymphoid differentiation (68). During adipogenesis, P-TEFb (containing CDK9_55_, a minor isoform of CDK9) (73) interacts with, and phosphorylates the peroxisome proliferator-activated receptor gamma (PPAR γ), the master regulator of adipocyte differentiation, to activate transcription of its target genes in pre-adipocytes (71). Furthermore, CDK9-cyclin T1 is required for neuronal differentiation induced by retinoic acid as indicated by increased expression of both CDK9 and cyclin T1 (69, 72). This neuronal differentiation is linked to increased expression of differentiation-associated genes as a result of retinoic acid inducing the interaction of P-TEFb with a transcription factor, called Zinc Finger MYND-Type Containing 8 (72).

### Role of P-TEFb in DNA Repair

While the biological role of the CDK9-cyclin T1 complex is well established, the function of CDK9-cyclin K has remained elusive for a long period of time. Although CDK9-cyclin K can phosphorylate RNAP II CTD *in vitro* (30), this complex can only activate transcription while tethered to RNA but not DNA (74). Insight into the role of CDK9-cyclin K emerged with the identification of cyclin K as a transcription target for p53 in response to DNA damage (75). The depletion of CDK9 and cyclin K, but not cyclin T, with RNAi was shown to impair the cell cycle in response to replication stress and DNA damage (76). Relative to transcriptionally mediated mechanisms, CDK9-cyclin K is presumed to play a direct role in the repair of damaged DNA by interacting with components of the ATM-and Rad3-related (ATR) pathways that respond to DNA damage, such as ATR, ATR interacting protein, and claspin (76). Furthermore, small hairpin RNA (shRNA)-mediated depletion of CDK9_55_ induces double strand DNA breaks and apoptosis (77). CDK9_55_ also interacts with Ku70, a key protein in nonhomologous end-joining, and might play a role in DNA repair. Interestingly, this interaction might involve cyclin K as Ku70 does not associate with either cyclin T1 or T2 (77).

## Regulation of P-TEFb Activity

The activity of P-TEFb is highly regulated because its function is important for the efficient expression of most genes (34). Numerous transcriptional, translational, and posttranslational mechanisms are employed to control the protein level and biological activity of P-TEFb (34).

### Regulation by Sequestering in an Inactive Complex

**Figure 6:**
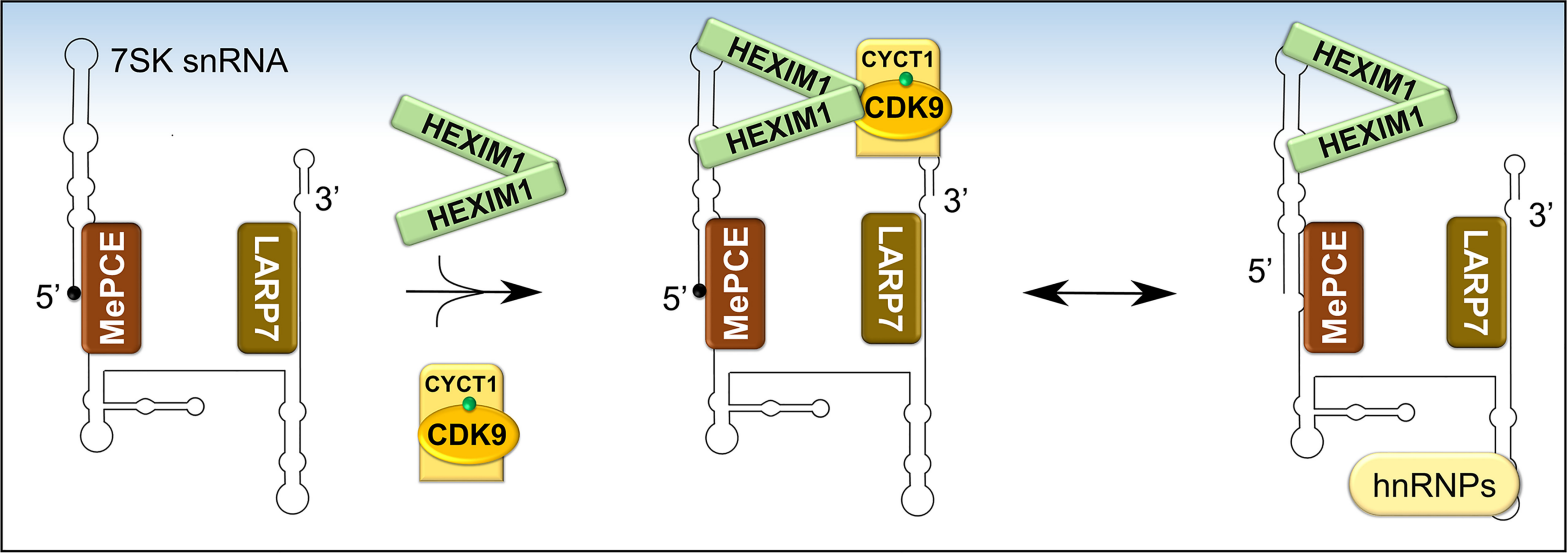
Formation and composition of 7SK snRNP. Following the folding of 7SK RNA into a four-stem loop structure, MePCE and LARP7 bind and protect its 5’ and 3’ ends, respectively, from catalytic degradation. One mechanism of protection involves capping of the 5’ end of 7SK RNA by MePCE (depicted as a black dot). The stable 7SK snRNP core then binds dimers of HEXIM1 which exposes their P-TEFb binding domains. Subsequently, HEXIM1 binds activated P-TEFb (CDK9 phosphorylated on Thr186, green dot) and this inhibits its kinase activity. During transcriptional activation, P-TEFb is released and 7SK snRNP is stabilized by binding to heterogeneous nuclear ribonucleoproteins (hnRNPs).

P-TEFb can integrate into two functionally opposite complexes, which are in equilibrium. More than half of cellular P-TEFb is reversibly sequestrated in a large inhibitory ribonucleoprotein complex (**Figure 5**), while the remainder is bound to BRD4 and transcriptionally active (27, 78). **Figure 6** shows in more detail the control over the level of inhibitory complex. P-TEFb is sequestrated by hexamethylene bisacetamide-inducible proteins (HEXIM 1 or 2) bound to a small, evolutionarily conserved nuclear RNA called 7SK snRNA (27, 78, 79). In this complex (hereafter named, 7SK snRNP), HEXIM is the main suppressor of P-TEFb function and was initially identified as a protein induced upon treating vascular smooth muscle cells with hexamethylene bisacetamide (78, 80). 7SK snRNA serves as an indispensable scaffold that mediates the interaction between HEXIM 1 or 2 and P-TEFb (**Figure 6**) (78, 81–83). Due to its vital role, the stability of 7SK snRNA is maintained by two proteins known as La related protein [LARP7 (84, 85)], and methyl phosphate capping enzyme [MePCE (85, 86)], which protect the 7SK snRNA from degradation by exonucleases at the 3’ and 5’ ends, respectively. The reversible sequestration of P-TEFb by 7SK snRNP plays a key role in maintaining a strict balance in the overall cellular gene expression (**Figures 5** and **6**).

**Figure 7:**
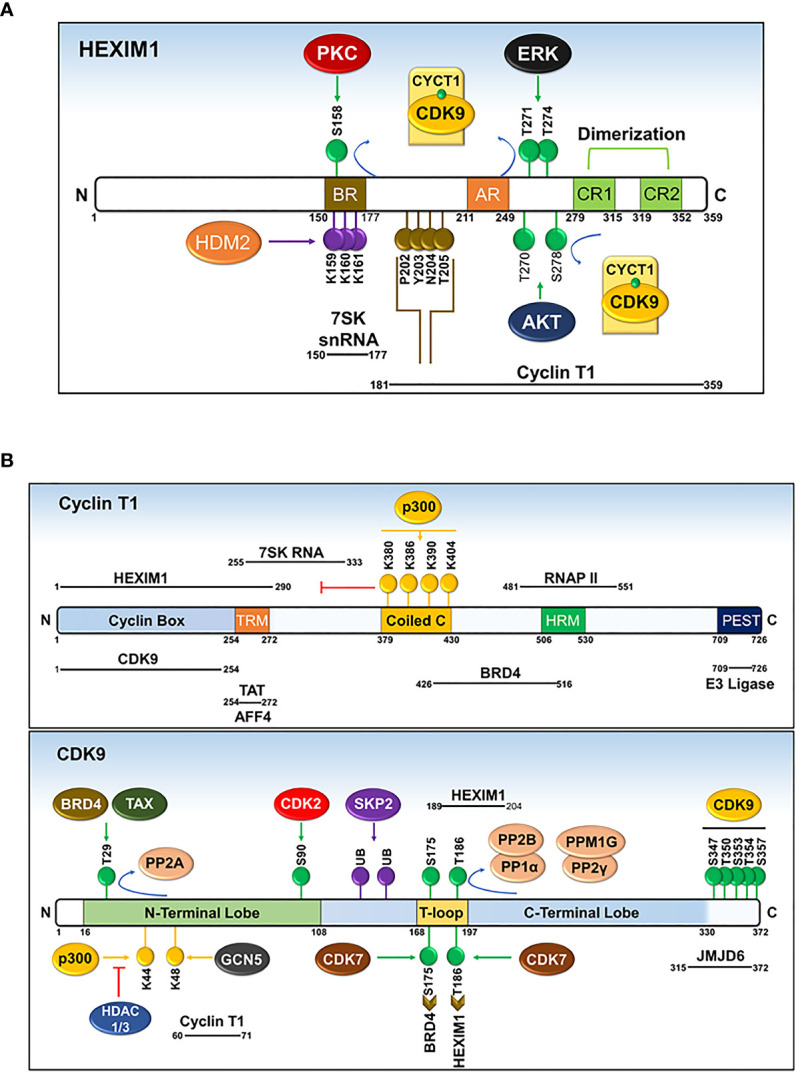
Domains and post-translational modifications of HEXIM1 **(A)**, cyclin T1 **(B)**, and CDK9 **(B)**. Phosphorylated (green), ubiquitinated (purple), acetylated (yellow), and PYNT (brown) motifs are shown. Numbers indicate the positions of amino acid residues. BR, basic region; AR, acidic region, encompassing AR1 and AR2; CR, coiled-coil region. Solid black lines with indicated spans of amino acid residues indicate the regions responsible for interactions with other binding partners. Modified from Cho, Schroeder and Ott, Cell Cycle, 9 (9), 1697-1705 (2010).

Prior to sequestering P-TEFb, HEXIM1 is found as a dimer formed through its two C-terminal coiled-coil (CR) regions (residues 279-352) and is incapable of interacting with and/or inhibiting P-TEFb (**Figures 6** and **7A**) (87, 88). Such inability emanates from an autoinhibitory electrostatic interaction between a highly basic region (BR, residues 150-177, KHRR motif) and two acidic regions (AR1 and 2, residues 211-249) of HEXIM1, conferring a conformation that opposes its interaction with P-TEFb (**Figure 7A**) (78, 89). Binding of the 5’ terminal hairpin of 7SK snRNA with the BR of HEXIM1 causes a conformational change in HEXIM1, unmasking its C-terminal P-TEFb binding domain (residues 181-359) for interaction with the cyclin box of cyclin T1 (88, 89). In this complex, cyclin T1 also makes contact with the 3’ hairpin of 7SK RNA (**Figure 7B**) (90). HEXIM1 inactivates CDK9 through its PYNT motif (residues 202-205), which masks the catalytic site of CDK9 and occupies its substrate recognition motif (**Figures 6** and **7A**) (88, 91). Interestingly, phosphorylation of CDK9 on its Thr186 residue is required for the sequestration of P-TEFb in the inhibitory complex (88, 92). This suggests that the inhibitory complex serves as a pool for transcriptionally active kinase, as Thr186 phosphorylation is critical for P-TEFb kinase activity. Because HEXIM homodimerizes, it is proposed that the 7SK snRNP complex comprises two HEXIM1 molecules, binding to 7SK snRNA and P-TEFb (88).

Orchestrating the function of P-TEFb to activate paused RNAP II complexes on some genes while sparing others requires specific delivery of P-TEFb. Previously, simple diffusion of 7SK snRNP was proposed as the mechanism for transporting P-TEFb to activated genes based on the finding that 7SK snRNP is readily extracted from the nuclei under non-harsh conditions (e.g., under low salt treatment) (93). Separate reports, however, have indicated the presence of 7SK snRNP on chromatin. This is based on the existence of inactive P-TEFb and components of the inhibitory complex (e.g. HEXIM1, LARP7, and 7SK snRNA) with the non-phosphorylated form of RNAP II in the pre-initiation complex (94). Coincidentally, subsequent evidence has shown the co-occupancy of HEXIM1, LARP7, and 7SK snRNA with RNAP II on the transcribed loci of a wide number of active protein-encoding genes and further identified a chromatin anchoring mechanism for 7SK snRNP (e.g., methylated histone (H4R3me) on specific enhancers, termed anti-pause enhancers) (95, 96). These observations indicate that 7SK snRNP is tethered to chromatin to selectively guide the function of a transcription-ready P-TEFb (**Figure 5**).

**Figure 8:**
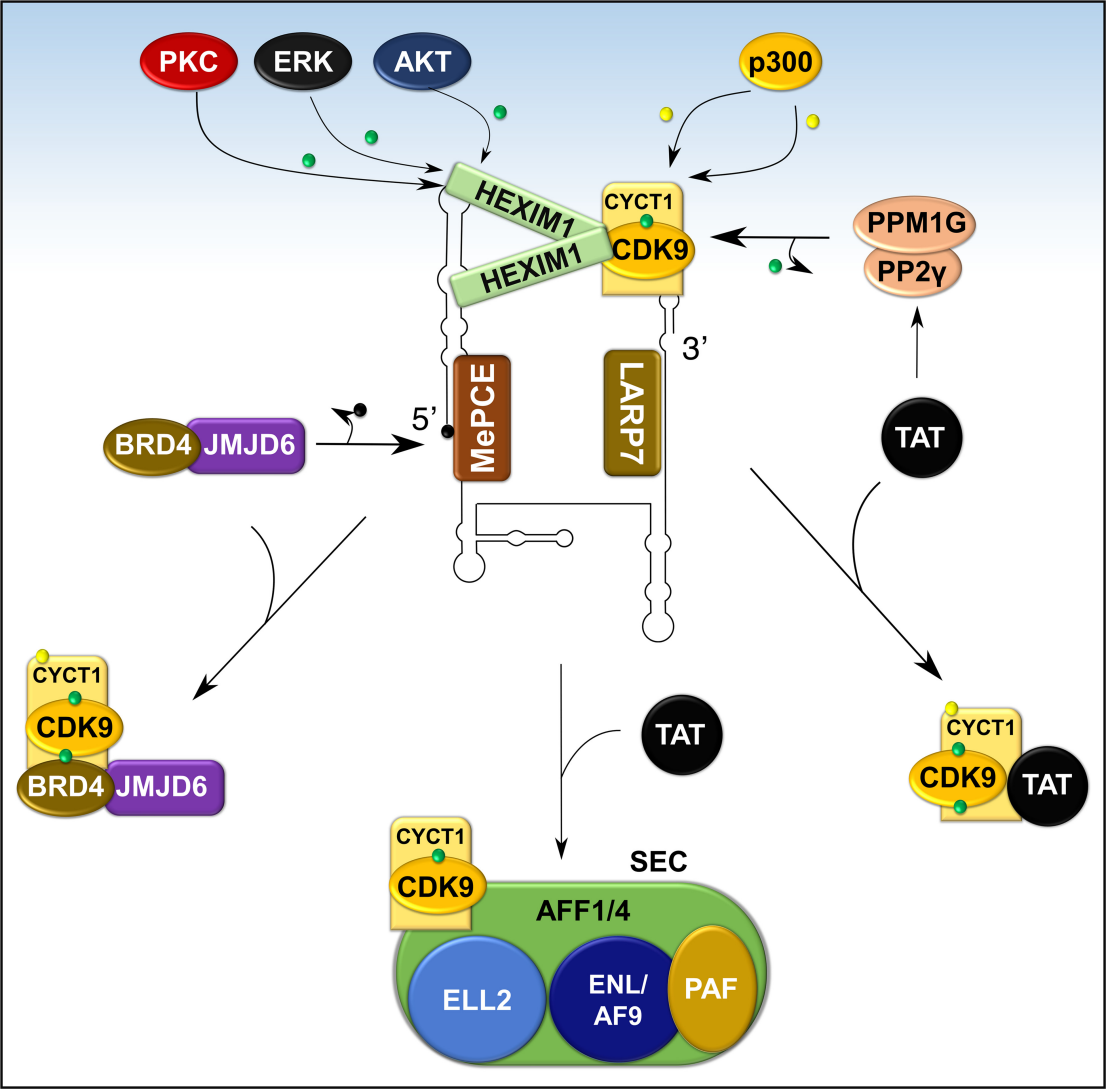
Recruitment of P-TEFb from 7SK snRNP. Various signaling pathways and stress conditions liberate P-TEFb from 7SK snRNP through PTMs of the components of 7SK snRNP (HEXIM1 and cyclin T1) or direct recruitment (BRD4, TAT, or super elongation complex, SEC). The major PTM involves phosphorylation (green dot) of HEXIM1 in various residues and acetylation (yellow dot) of cyclin T1. TAT recruits P-TEFb by collaborating with phosphatases (PPM1G and PP2γ) which dephosphorylate CDK9 on Thr186. Meanwhile, BRD4 interacts with JMJD6, a histone demethylase, which demethylates (black dot) the 5’ end of 7SK RNA and destabilizes the inhibitory core. AFF1/4, ALL-fused gene from chromosome 1/4 family member; ELL2, Eleven-nineteen lysine-rich in leukemia; ENL, Eleven nineteen leukemia; AF9, ALL-fused gene from chromosome 9.

The precise molecular mechanism for the liberation of P-TEFb from 7SK snRNP in response to cellular signals or stress conditions (e.g., UV, actinomycin D) remains to be elucidated but might involve post-translational modifications of the components of 7SK snRNP or direct recruitment by transcription regulators (e.g. BRD4, HIV TAT) (**Figures 7A, B** and **8**). Multiple modifications directed towards HEXIM1, CDK9, and cyclin T1 induce the release of P-TEFb. For instance, T-cell receptor signaling disrupts 7SK snRNP and activates P-TEFb function *via* phosphorylation of HEXIM1 mediated by protein kinase C (PKC, on Ser158) and extracellular-signal-regulated kinase (ERK, on Tyr271, 274) (**Figure 7A**) (97, 98). In a different cellular context (e.g. HIV infected cells), hexamethylene bisacetamide (HMBA) disrupts 7SK snRNP by activating the Phosphatidylinositol 3-kinase (PI3K)/Protein Kinase B (AKT) pathway, which inhibits the interaction between HEXIM1 and P-TEFb by phosphorylating HEXIM1 on Thr270 and Ser278 (**Figures 7A** and **8**) (99). In addition, HMBA and UV light activate Ca^+2^-dependent phosphatases (PP2B and PP1α) which cause dephosphorylation of CDK9 on Thr186, releasing P-TEFb (100). Besides these phosphorylation-dephosphorylation mechanisms, acetylation of cyclin T1 on its C-terminal lysine residues (Lys, K380, 386, 390 and 404) by p300 further contribute to the release of P-TEFb (**Figures 7B** and **8**) (101).

In addition to intracellular signaling, transcriptional factors can also directly release P-TEFb from 7SK snRNP. The HIV1 TAT activates HIV transcription by hijacking P-TEFb from 7SK snRNA through a high-affinity competition with HEXIM1 for interaction with cyclin T1 (102). To complement this direct effect, TAT also recruits protein phosphatases PPM1G/PP2Cγ to the HIV promoter to dephosphorylate CDK9 on Thr186 to enhance the release of P-TEFb (**Figure 8**) (96). In normal cellular transcription, the release of P-TEFb from 7SK snRNP depends on BRD4 (27, 103). BRD4 is a ubiquitously expressed nuclear protein that recognizes acetylated histone during active transcription (**Figure 5**) and serves as an adaptor for recruiting key transcription factors (104). During active transcription, acetylated histone-bound BRD4 recruits an arginine histone demethylase, JMJD6 (105), to chromatin-anchored 7SK snRNP on anti-pause enhancers (A-PE). Here, JMJD6 releases P-TEFb from 7SK snRNP by demethylating both H4R3me and the 5’ hairpin of 7SK RNA, which dissociates 7SK snRNP from chromatin and exposes 7SK RNA for degradation (**Figure 8**) (95). Once released, both BRD4 and JMJD6 interact with respective cyclin T1 and CDK9 (**Figures 7A, B**), through their P-TEFb interacting domains [PID; BRD4, residues 1209-1362 and JMJD6, residues 1-305 (95, 106)], and deliver P-TEFb to poised RNAP II.

A small fraction of active P-TEFb can also be found in the Super Elongation Complex (SEC), a multicomponent, potent transcription activator (**Figure 8**). The components of SEC are known fusion partners for Mixed Lineage Leukemia (MLL) and this multiplex is actively recruited by TAT during HIV1 replication (107, 108). Components of SEC such as AF9, AFF1/4, ENL, ELL interact directly with P-TEFb (also see section on hematological malignancies below) and this interaction is increased by TAT. Besides BRD4 or SEC, other transcription factors such as NFκB might directly or indirectly deliver P-TEFb to target genes (96, 107).

Once P-TEFb is extracted by BRD4/JMJD6 or other transcription factors, 7SK snRNA is stabilized by binding to heterogeneous nuclear ribonucleoproteins (hnRNPs A1, A2/B1, R, and Q; **Figure 6**) (109, 110). The exact mechanism for the reassembly of the 7SK snRNP is not known but it is proposed that upon termination of transcription, hnRNPs are recruited by the nascent mRNA, thereby releasing 7SK snRNA to reassemble with P-TEFb and HEXIM1 (109, 110).

### Regulation of P-TEFb by Post-Translational Modifications

Besides sequestration in an inhibitor complex, the biological activity of P-TEFb is further controlled by post-translational modifications directed toward CDK9 and cyclin T1. These modifications, which include phosphorylation, acetylation, and ubiquitination of CDK9 and/or cyclin T1, increase or decrease the activity of P-TEFb.

#### Regulation by Phosphorylation

Among the various modifications documented, phosphorylation of several Ser and Thr residues of CDK9 and its cyclin T1 partner plays a key regulatory function (**Figure 7B**). Phosphorylation of a conserved Thr186 residue in the T-loop structure of CDK9 (**Figure 2**) is necessary for its enzymatic activity (88, 92). This phosphorylation triggers a conformational change in the CDK9-cyclin T1 heterodimer exposing the ATP and substrate binding sites (22). Moreover, as described above, binding of P-TEFb to the components of 7SK snRNP is also dependent on the phosphorylation of CDK9 at Thr186 (pThr186), indicating that the inhibitory complex serves as a pool for efficient and prompt release of active P-TEFb in response to stress conditions (92, 100). Initially, autophosphorylation was described as the main mechanism for the formation of pThr186, based on an *in vitro* kinase assay employing purified CDK9-cyclin T1 complex (22). Recent evidence, however, revealed that CDK7, a CDK-activating kinase (CAK) for various cell cycle CDKs, is responsible for forming pThr186 (111). Besides CDK7, a global search for other kinases responsible for forming pThr186 indicated that siRNA knockdown of Ca^2+^/calmodulin-dependent kinase 1D decreases the level of pThr186, although a direct link for this role was not established (112).

Once P-TEFb dissociates from the inhibitory complex, CDK9 is phosphorylated on a second highly conserved T-loop residue, Ser175 (**Figure 7B**) (103). CDK9 carrying pSer175 is found exclusively outside of the 7SK snRNP complex and this phosphorylation step promotes the binding of P-TEFb with BRD4 and/or TAT (103, 113). It was proposed that pSer175 induces a conformational change in P-TEFb favoring the interaction of cyclin T1 with BRD4 (103). Recently, an *in vitro* kinase assay and cellular experiments employing THZ1, a highly selective covalent CDK7 inhibitor, have identified CDK7 (as part of CAK) as the kinase that phosphorylates CDK9 on Ser175 (114). The direct contribution of pSer175 to the kinase activity of CDK9 is not clearly established. This comes from various findings showing that a mutation of Ser175 to alanine renders CDK9 inactive in an *in vitro* kinase assay (88), while at the same time, it increased TAT-dependent HIV1 transcription at the cellular level (113). Furthermore, mutation of Ser175 to aspartic acid, mimicking the phosphorylated Ser, failed to activate both CDK9 kinase activity *in vitro* (88) and TAT-dependent HIV1 transcription *in vivo* (113).

The activity of CDK9 is reduced by phosphorylation of an N-terminal Thr29 (115), a modification that is homologous to inhibitory phosphorylation of CDK2 on Thr15 (116). Surprisingly, BRD4 and TAX [transactivator of Human T-lymphotropic Virus Type 1 (HTLV-1)], which recruit P-TEFb for basal and HTLV-1 transcriptions, respectively, induced phosphorylation of CDK9 on Thr29 (**Figure 7B**) (115, 117). This phosphorylation mainly occurs in the pre-initiation complex, following recruitment of CDK9 by either BRD4 or TAX, and is necessary for limiting the function of CDK9 during initiation of transcription (117). Following succession from transcription initiation to elongation, PP2A dephosphorylates pThr29 (**Figure 7B**) and activates the positive transcription elongation activity of CDK9 (117).

Additional phosphorylation of CDK9, vital mainly for the activation of HIV1 replication, includes phosphorylation of a cluster of C-terminal Ser/Thr residues (Ser347, Ser353, Ser357; Thr350, Thr354) and an N-terminal Ser90 residue (**Figure 7B**) (118–120). Autophosphorylation of the C-terminal residues of CDK9 and cyclin T1 increases the binding of the TAT-P-TEFb complex to the TAR RNA (118, 119). In addition, phosphorylation of CDK9 on Ser90 by CDK2 positively contributes to HIV1 replication (120). Besides the above-mentioned residues, several other phosphorylated residues of CDK9 with unknown function, were identified by mass spectrometry (121).

#### Regulation by Acetylation

Acetylation of both CDK9 and cyclin T1 is another important post-translational modification that regulates the biological function of P-TEFb (**Figure 7B**). Namely, acetylation of four lysine residues within the coiled-coil region of cyclin T1 (Lys380, Lys386, Lys390, and Lys404) induces the dissociation of P-TEFb from 7SK snRNP and is found exclusively in the active P-TEFb complex (101). Acetylation of these residues is mediated by the histone acetyl-transferase (HAT), p300, which liberates P-TEFb from HEXIM1 and 7SK snRNA (**Figures 7B** and **8**) (101). Consequently, acetylated cyclin T1 binds the second bromodomain (BDII) of BRD4 and supports the recruitment and transcriptional activity of P-TEFb (122). In contrast to normal cellular transcription, the HIV1 TAT protein has evolved the ability to recruit the non-acetylated cyclin T1 directly from the inhibitory complex to activate HIV1 transcription (102).

In addition to cyclin T1, CDK9 is also acetylated on two N-terminal lysine residues, namely Lys44 and Lys48, by p300 and GCN5 (**Figure 7B**) (123, 124). While p300 and GCN5 acetylate both lysine residues, Lys44 is the preferred site for p300 while GCN5 mainly targets Lys48 (123, 124). Contrary findings have been reported regarding the outcome of CDK9 acetylation on its kinase and transcriptional activity (123, 124). On the one hand, Fu et al.** reported that acetylation of CDK9 on Lys44 increases its activity as the mutation of Lys44 to Arginine or overexpression of histone deacetylases (HDAC1 and 3, directed towards Lys44) markedly impaired CDK9’s kinase and transcriptional activities (123). On the other hand, Sabo et al. showed that acetylation of CDK9 inhibits the binding of ATP to CDK9 and thus hinders its kinase activity (124). Recently, the latter finding was supported by a study showing that by counteracting GCN5-mediated Lys48 acetylation with sirtuin7, a nicotinamide adenine dinucleotide (NAD)-dependent deacetylase, there was an increased transcriptional activity of P-TEFb (125).

#### Regulation by Ubiquitination

Polyubiquitination of CDK9 and its negative regulator HEXIM1 provides an additional mechanism for the regulation of P-TEFb (**Figures 7A, B**) (29, 126). Recruitment of the SCF E3 ubiquitin ligase core components (S-phase kinase-associated protein 1 (SKP1), cul-1, and p45^SKP2^) by cyclin T1 through its C-terminal PEST domain (residues 709-726) mediates polyubiquitination and subsequent degradation of CDK9 by proteasomes (29). Since the protein level of CDK9 does not change in a manner akin to kinases regulating the cell cycle (59), the functional relevance of proteolytic degradation of CDK9 to its regulation is not clear. Interestingly, contrary to the well-known function of ubiquitination, HIV1 transactivation by TAT is increased by the ubiquitination of CDK9 which facilitates the formation of a ternary complex between P-TEFb, TAT, and TAR RNA (127). TAT also recruits the UBE2O ubiquitin ligase in the cytoplasm to ubiquitinate HEXIM1 in a non-degradative manner (128). This ubiquitination step releases HEXIM1 from 7SK snRNP and liberates P-TEFb for transport from the cytoplasmic pool to the nucleus (128). Similarly, ubiquitination of HEXIM1 by human double minute-2 protein (HDM2, **Figure 7A**), a p53-specific E3 ubiquitin ligase, does not lead to proteasome-mediated degradation, but instead increases its sequestering and thus inhibition of P-TEFb, suggesting a role of ubiquitination beyond proteasome-mediated degradation (126).

## The Role of P-TEFb in Cancer

A plethora of genetic aberrations have been discovered as underlying causes for blood and solid cancers. Despite the overwhelming amount of known cancer-causing mutations, most tumors are reliant on continuously activated gene expression. Therefore, it does not come as a surprise that many studies have been conducted to highlight the link between P-TEFb and most known types of cancer. Here, we first focus comprehensively on blood cancers since the rationale for the use of CDK9 inhibitors can be best supported with known genetic aberrations and gene mutations underlaying these diseases. We also highlight the potential role of P-TEFb in solid tumors using breast, prostate, and hepatocellular cancers as examples. **Table 1** provides a summary of the studies providing links between P-TEFb and a range of other cancers as well as those discussed in more detail below.

**Table 1:** Studies that have described links between P-TEFb and different cancers.

Origin	Cancer Type	Potential Mechanistic Link and Biomarkers	Method Used	Ref.
Blood	Acute myeloid leukemia	MLL	shRNA Pharmacological inhibition	(129–132)
		MCL-1	Pharmacological inhibition*	(133, 134)
		HEXIM1	BRD4 pharmacological inhibition	(135)
	Chronic lymphocytic leukemia	BCL-2, MCL-1	siRNA Pharmacological inhibition	(136, 137)
	Acute lymphoblastic leukemia	MCL-1, XIAP	Pharmacological inhibition	(138)
	Diffuse large B-cell lymphoma	MYC, MCL-1	Genetic knockdown Pharmacological inhibition*	(139, 140)
	Burkitt’s lymphomas	MYC, MCL-1	Pharmacological inhibition*	(141, 142)
	Adult T-cell leukemia/lymphoma	MYC, MCL-1	Pharmacological inhibition*	(143)
	Multiple myeloma	MCL-1	Pharmacological inhibition*	(144, 145)
	Aggressive natural killer cell leukemia	MCL-1	Pharmacological inhibition*	(146)
	Peripheral T-Cell lymphomas		Pharmacological inhibition	(147)
	Mantle cell lymphoma	MCL-1	Pharmacological inhibition	(148)
Bone	Osteosarcoma	BIRC5, MCL-1	siRNA Pharmacological inhibition*	(149, 150)
Brain	Neuroblastoma	N-MYC, CDK9	mRNA expression shRNA Pharmacological inhibition	(69, 151, 152)
	Medulloblastoma	MYC, cyclin D1, BCL-2, CDK9	Immunohistochemistry Pharmacological inhibition*	(153)
Breast	Estrogen receptor positive	BCL-2, cyclin B1, cyclin E1	siRNA CRISPR/Cas9 Pharmacological inhibition	(154–156)
	Triple-negative breast cancer	MYC, MCL-1, cyclin B1, CDK9	CRISPR/Cas9 Pharmacological inhibition*	(156, 157)
Female reproductive organs	Ovarian cancer	MCL-1, BAX, CDK9	Immunohistochemistry siRNA, shRNA Pharmacological inhibition*	(158, 159)
	Cervical cancer	AKT2, P53	siRNA	(160)
Gastrointestinal	Hepatocellular cancer	MYC	shRNA Pharmacological inhibition	(161)
	Pancreatic cancer	KRAS mutant, MYC, CDK9	Immunohistochemistry Pharmacological inhibition*	(162–164)
	Esophageal cancer	MCL-1, AXL	Pharmacological inhibition*	(165, 166)
	Colon cancer	MCL-1, MYC, cyclin D1	shRNA Pharmacological inhibition	(167)
Lung	Non-small cell lung cancer	c-FLIP, MCL-1	siRNA Pharmacological inhibition	(168–170)
	Small cell lung cancer	MYC	Pharmacological inhibition*	(171)
Male reproductive organs	Prostate cancer	AR signaling	BRD4 pharmacological inhibition	(172)
Skin	Melanoma		Pharmacological inhibition	(173)
Others	NUT midline carcinoma	MYC, MCL	shRNA Pharmacological inhibition*	(174)
	Head and neck squamous cell carcinoma	Cyclin D1	siRNA Pharmacological inhibition	(175)

*Selective Inhibitors: BAY1143572; AZ5576; NVP-2; LDC000067; CDK9i.

### Hematological Malignancies

#### P-TEFb in Leukemia

**Figure 9:**
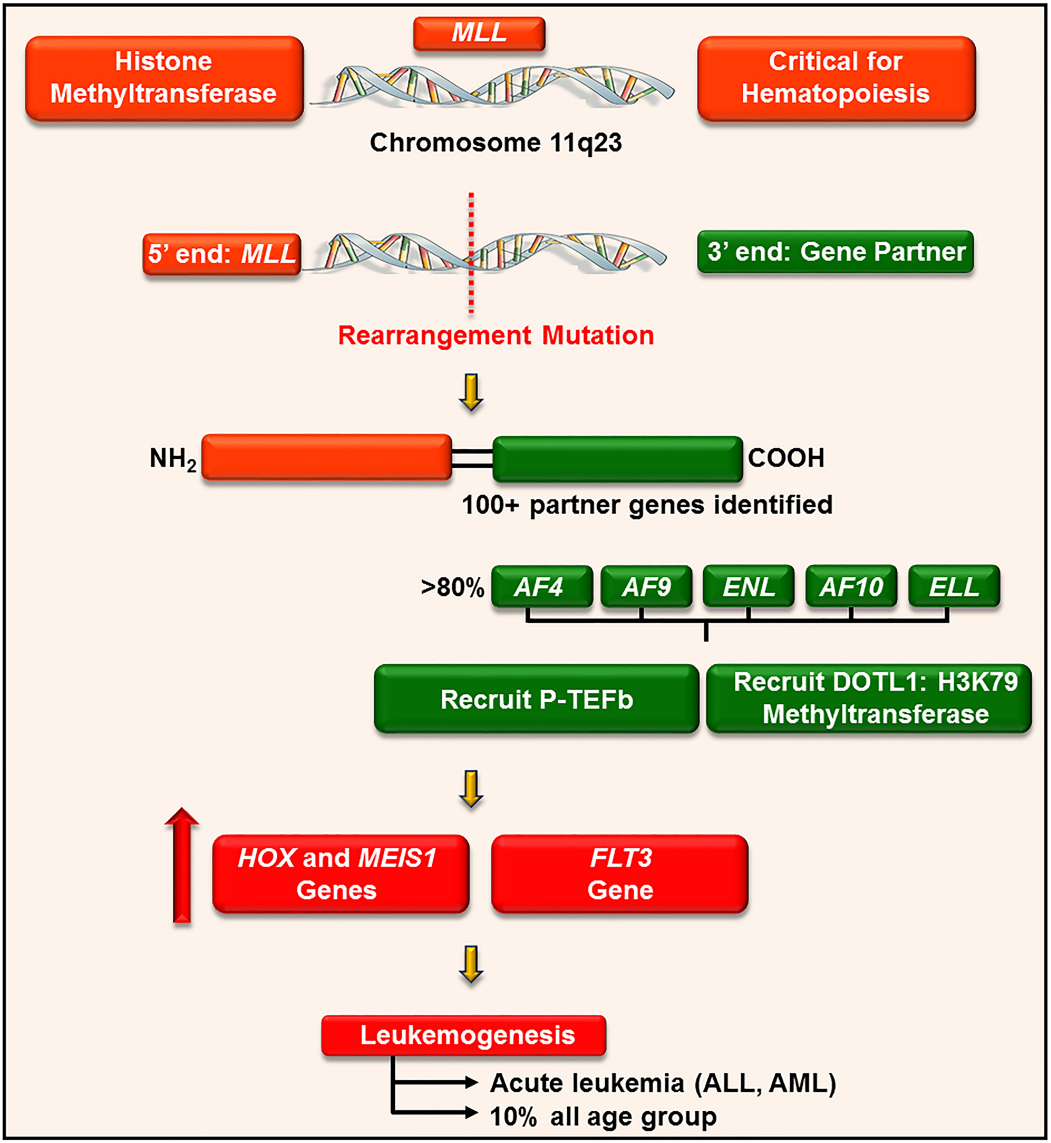
P-TEFb is required for the MLL transcription program and leukemogenesis. MLL is a histone methyltransferase ubiquitously expressed in hematopoietic progenitor cells and plays a key role in their self-renewal. For unknown reasons, *MLL* gene (on chromosome 11q23) undergoes a trans-locational mutation where its 5’ end is fused with the 3’ end of numerous genes. The majority of these partner genes are nuclear transcription factors that recruit P-TEFb and DOTL-1 leading to upregulated expression of *HOX*, *MEIS1*, and *FLT3*. These proteins drive leukemogenesis by blocking differentiation and driving active proliferation.

P-TEFb plays a well-recognized role in the pathogenesis of many hematological malignancies, such as leukemia. This is particularly true in leukemia harboring a chromosomal translocationw mutation on chromosome 11q23 (176). This loci encodes for a histone 3 lysine 4 methyltransferase protein, called mixed-lineage leukemia (MLL) (177). MLL is ubiquitously expressed in myeloid and lymphoid progenitor cells and increases the expression of a cluster of HOXA homeobox genes (e.g. *HOXA7* and *9*) and the gene for the HOX-dimerization partner, *MEIS1* (177). These genes control self-renewal of hematopoietic stem cells and are downregulated during hematopoietic differentiation (178). For reasons not clearly defined, chromosome 11 frequently undergoes an in-frame translocation mutation at the locus 11q23, where the 5’ end of *MLL* (containing its target gene binding motifs) is fused with the 3’ end of a wide variety of unrelated partner genes, generating chimeric MLL fusion proteins (**Figure 9**) (179). These fusion proteins are aberrant transcription factors that increase the expression of *HOXA* and *MEIS1* genes, leading to a pre-leukemic state by blocking hematopoietic differentiation (177). The actively proliferating pre-leukemic progenitor cells are highly susceptible to secondary mutations (e.g., mutation in the fms-like tyrosine kinase (FLT3) receptor) which aids in their transformation into acute leukemia (180).

MLL rearrangement leukemia has a poor prognosis and accounts for approximately 10% of all cases of acute leukemia in humans, irrespective of age. Specifically, MLL fusion is associated with > 70% of infantile acute lymphoblastic leukemia (ALL), 35 – 50% of infant acute myeloid leukemia (AML), and 1-15% of therapy-related leukemia (e.g. patients treated with topoisomerase II inhibitors) (177).

MLL is involved in more than 100 different rearrangements and 64 translocation partner genes have been identified (**Figure 9**) (179). Despite a large number of partners, only nine proteins account for more than 90% of MLL rearrangements, namely AF4, AF9, ENL, AF6, AF10, ELL, AF1p, AF17, and SEPT6 (179). The majority of these translocation partners collectively associate to form a large macromolecule called ENL-interacting proteins or elongation-assisting proteins (EAP) (181). EAP consist of three major components (1): SEC (e.g. AF4, AF9, ENL, and AFF4) which recruits P-TEFb and other elongation factors (e.g. ELL1-3, **Figure 8**) to activate transcription elongation (2, 176), Dot1-containing complex (DotCom) which methylates lysine 79 on histone 3 through the activity of disruptor of telomeric silencing 1-like (DOT1L) (182), and (3) MLL fusion partner ENL, which forms a scaffold for the multiprotein complex and inhibits negative regulators of MLL (e.g. polycomb proteins) (183). These combined effects lead to continuous ectopic expression of *HOXA* genes and finally contribute to leukemogenesis. In line with these roles, pharmacological inhibition of CDK9 (by flavopiridol, dinaciclib, and CDKI-73) significantly delay disease progression and improve survival in mouse models of MLL-ENL and MLL-AF9, respectively (129, 130, 184).

Leukemia harboring MLL fusions possess a high level of expression and frequent activating mutations in the *FLT3* gene (185, 186). FLT3 is a membrane-bound receptor tyrosine kinase that regulates the survival, proliferation, and differentiation of hematopoietic stem cells (187). Stimulation of FLT3 by its cognate ligand activates signal transduction networks, mainly through PI3K/AKT and mitogen-activated protein kinase (MAPK) pathways (187). *FLT3* is mutated and constitutively active in 30% of AML, and the most common mutation, known as internal tandem duplication (ITD), involves an in-frame duplication of short sequences in the juxtamembrane domain of the receptor. ITD thwarts an auto-inhibitory mechanism built into the FLT3 wild type (WT) receptor leading to dimerization and constitutive activation of its signaling despite the absence of a ligand (187). Moreover, the co-occurrence of MLL fusion (MLL-AF9) and FLT3-ITD was found to accelerate the onset of AML in a mouse model, suggesting cooperation of the two oncogenes in leukemogenesis (188). This highlights the role played by P-TEFb for the transcription of MLL-fusion genes in leukemic cells harboring both MLL fusion and FLT3-ITD.

Besides its role in MLL, P-TEFb is also involved in the pathogenesis of AML and chronic lymphocytic leukemia (CLL) through its direct role in myeloid cell leukemia 1 (MCL-1) transcription. High expression of MCL-1 is responsible for the development and survival of AML cells (189). Evaluation of clinical AML samples (e.g. leukemic blasts and primary human hematopoietic subsets) indicated high MCL-1 expression (190). In addition, MCL-1 is also upregulated in 50% of recurrent AML cases and is linked with a poor prognosis and/or response to chemotherapy (191). The continuous survival state of leukemic blasts mediated by high MCL-1 expression requires constant activation of P-TEFb due to the short half-life of MCL-1 (133, 134). Therefore, CDK9 inhibition dramatically reduces MCL-1 expression in AML and produces strong anti-leukemic activity in AML animal models (134, 192). These observations have led to the current clinical trials investigating the value of CDK9 inhibitors (e.g., BAY1143572 and CDKI-73) for AML treatment (193).

CLL is the most common leukemia in elderly patients and is characterized by the accumulation of mature but functionally impotent B-lymphocytes in blood, bone marrow, and lymph nodes (194). Relative to other hematological malignancies, CLL is a non-proliferative form of leukemia associated with constitutive activation of the B-cell receptor signaling pathway and overexpression of the B-cell lymphoma 2 (BCL-2) family of anti-apoptotic proteins (e.g., BCL-2, MCL-1) (195, 196). The dependence of CLL on P-TEFb-mediated transcription was confirmed by the apoptotic effects on CLL cells of inhibiting CDK9 by pharmacological (e.g., flavopiridol, dinaciclib, and CDKI-73) and biological means (siRNA) (136, 197, 198). This reliance was further established by the strong anticancer effect of both flavopiridol and dinaciclib in refractory CLL patients in phase II/III clinical trials (137, 199–201).

#### P-TEFb in Other Hematological Malignancies

P-TEFb has also been implicated in the development of lymphoma, particularly diffuse large B-cell lymphoma. This is a non-Hodgkin lymphoma characterized by malignant and diffuse proliferation of large B lymphocytes (202). Dysregulation of c-MYC is essential in its pathogenesis and involves either a c-MYC rearrangement mutation, most commonly involving its translocation into heavy- or light-chain immunoglobulin loci (5-15%), or gene amplification (30-50%). These changes often confer aggressive clinical courses (202). As a general transcription factor, MYC directly interacts and recruits P-TEFb to its promoter and other target genes to mediate RNAP II pause release (35). Inhibition of CDK9 by selective inhibitors (e.g., AZ5576) or by genetic knockdown negatively regulated MYC and MCL-1 expression, and induced apoptosis in primary and transformed cells from this lymphoma, providing an attractive therapeutic strategy (139, 140).

Furthermore, dysregulation of MYC and MCL-1 transcription is associated with the development of adult T-cell leukemia/lymphoma, an aggressive proliferation of mature T lymphocytes transformed by HTLV-1 (143). Similar to diffuse large B-cell lymphoma, cells from this leukemia/lymphoma are highly susceptible to the apoptotic effect of selective pharmacological inhibition of CDK9 (143). Other rare hematological malignancies such as multiple myeloma, aggressive natural killer cell leukemia, and peripheral T-Cell lymphomas have also been found responsive to CDK9 inhibitors (144, 146, 147).

### Solid Cancers

#### P-TEFb in Breast Cancer

There are three major subtypes of breast cancer, based on the presence or absence of receptors for estrogen (ER+/-), progesterone (PR+/-) and human epidermal growth factor receptor 2 (HER2+/-). The majority of patients (70%) are diagnosed with ER+/PR+/HER2- cancers, followed by HER2+ (15-20%), and ER-/PR-/HER2- triple-negative (15%) cancers (203). These subtypes have different risk profiles, treatment strategies, and clinical outcomes (203). Besides this classification, genetic profiling has been used to identify different breast cancer subtypes having diverse clinical courses (204). Regardless of these genetic heterogeneities, breast cancers are reliant on continuously activated gene expression programs that are dependent on P-TEFb. For instance, the survival and proliferation of ER+ breast cancers are dependent on overexpression of the MYB proto-oncogene, a transcription factor which is a direct target for ER signaling, and regulator of BCL-2, cyclin B1, and cyclin E1 (154, 205). MYB transcription is regulated by ER-mediated recruitment of P-TEFb to transcription pause sites (205). In addition, breast cancers have evolved the capability of downregulating a tumor-suppressive microRNA (miRNA), miR-874, which suppresses proliferation by downregulating CDK9 expression (155). Apart from a direct mechanistic role, P-TEFb-mediated overexpression of MYC is associated with ER-independent growth in breast cancers resistant to hormone therapy (206). These roles are reinforced by the induction of apoptosis and inhibition of cell growth in both hormone therapy-sensitive and -resistant ER+ breast cancer cell lines (e.g., MCF-7) as a consequence of CDK9 inhibition by biological or pharmacological means (154, 155, 206).

Relative to ER/PR+ and HER2+ breast cancers, triple-negative breast cancers (TNBCs) are more aggressive subtypes with a higher frequency of relapses (207). The high level of inherent genetic heterogeneity in TNBCs and consequent lack of unifying molecular alterations has created a challenge for targeted therapy (207). Despite this heterogeneity, however, the gene expression profiles of TNBCs display a uniform trait of activated transcription of a cluster of TNBC-specific genes (156). These clusters are of transcriptional regulators and signal transducers, such as epidermal growth factor receptor (EGFR), Fos-related antigen 1, Forkhead box C1, MYC, and SOX9. Most are associated with large enhancer regions occupied by multiple transcriptional factors, called super-enhancers. As a result, these genes are exceptionally active and sensitive to inhibitors targeting key regulators of transcription (e.g., CDK7 by THZ1 and CDK9 by BAY1143572 and dinaciclib) (156, 157, 208). Clinically, high CDK9 expression in TNBC patients renders comparatively poor overall survival (157).

#### P-TEFb in Prostate Cancer

Androgen receptor (AR) signaling regulates the expression of genes vital for the growth, differentiation, and survival of prostate cells (209). The AR interacts directly with P-TEFb or its recruiter protein, BRD4, to mediate expression of its target genes (e.g., prostate-specific antigen, PSA) (172, 210). Moreover, P-TEFb phosphorylates AR on Ser81 and influences its chromatin binding, nuclear localization, and transcriptional activity (211, 212). These functions are overtly activated in prostate cancer cell lines and primary clinical samples and abrogated by non-selective CDK9 inhibitors (e.g., flavopiridol, roscovitine) (212–214).

Androgen deprivation has been the mainstay of treatment for advanced prostate cancers, but inevitably the disease relapses leading to a castration-resistant form (CRPC). In these phenotypes, AR signaling is still maintained *via* diverse mechanisms such as AR amplification or constitutively active AR splicing variants (215). Several lines of evidence point to P-TEFb involvement in AR signaling of CRPC. A group of AR-regulated enhancer RNAs (small non-encoding RNAs) are upregulated and interact with P-TEFb to promote the growth of CRPC (216). In addition, BRD4 interacts directly with and recruits the AR to target genetic loci that drive the proliferation of CRPC (172). Correspondingly, MYC, a critical downstream transcriptional target of BRD4/P-TEFb, was shown to be responsible for the overexpression of full-length AR and AR splice variants in CRPC patient samples (217). Besides MYC, the upregulation of MCL-1 in CRPC is responsible for the androgen-independent survival of CRPC (218). As an outcome, inhibition of the dysregulated AR transcription in CRPC through specific BRD4 inhibitors (e.g., JQ1) produces strong suppression of cellular proliferation (172, 219), although the direct consequence of P-TEFb inhibition in CRPC has not been established yet.

#### P-TEFb in MYC-Dependent Hepatocellular Cancer

Hepatocellular cancer is an aggressive, highly lethal (< 1-year survival rate in the advanced stage), and frequent type of primary liver cancer which originates from a series of genetic and epigenetic events following chronic liver diseases (220). Genomic studies identified MYC amplification and TP53 inactivation as frequent genetic alterations (221, 222). Gene silencing using a shRNA library identified the requirement of CDK9 for the sustained proliferation of hepatocellular cancer cells and their dependence on MYC (161). This finding was reinforced by the antitumor effects arising from shRNA-mediated and pharmacological (e.g., PHA-767491) inhibition of CDK9 in murine and human cell lines driven by MYC. Furthermore, the silencing of CDK9 inhibited MYC-dependent liver tumorigenesis in a mouse model and suppressed the proliferation of xenografts of murine and human hepatocellular cancer cells (161). A high level of CDK9 expression concurrent with a downregulation of miRNA-206, an inhibitor of translation from CDK9 mRNA, was noted in hepatocellular cancer cell lines (223).

## Inhibitors of CDK9 as Therapeutic Agents for Cancer

The discovery of flavopiridol as the first clinical CDK inhibitor, launched a race for the discovery of alternative small molecules with more potent and selective CDK9 inhibition, and some have entered clinical trials for treating solid and hematological malignancies. These inhibitors are competitive at the highly conserved catalytic ATP binding site (224), and as a consequence, they tend to target multiple CDKs and/or protein kinases, rendering them less attractive for use as therapeutic agents and chemical probes (225). Nevertheless, several inhibitors have been developed with improved selectivity towards CDK9, but only limited data are available regarding their broader selectivity profile and pharmacological properties.

### First Generation CDK9 Inhibitors

Flavopiridol (Alvocidib) was the first pan-CDK inhibitor to enter clinical trials with half maximum inhibitory values (IC_50_) below 400 nM against CDKs 1, 2, 4, 6, 7, and 9 (136, 226, 227). Initially, the anticancer mechanisms were attributed to the arrest of cells at the G_1_ and G_2_/M phases of their cycle through inhibition of CDK4/6 and CDK1, respectively. Later, the primary mechanism of action was ascribed to downregulation of cell cycle- and apoptosis-related genes *via* inhibition of CDKs 7 and 9 (136, 228). Flavopiridol showed inadequate efficacy relative to its toxicity when tested clinically against various solid and hematological malignancies, either as a single agent or in combination with other anticancer agents. Regardless of these outcomes, timed sequential combinations with cytarabine and mitoxantrone reached complete remission (CR) rates of 36-68% in relapsed/refractory (R/R) AML and newly diagnosed poor-risk AML (229). As a result, orphan drug designation has been assigned for the treatment of AML patients.

Seliciclib (roscovitine/CYC202) was the second pan-CDK inhibitor to enter clinical trials. It inhibited CDK9 with lower potency compared to flavopiridol (IC_50_ = 950 *vs.* 7 nM, respectively), but with improved selectivity (230). Seliciclib was more potent against CDKs 2, 5, and 7 (IC_50_ = 100, 160, and 490 nM, respectively) than CDK9 (230), and demonstrated anticancer activities in numerous preclinical cancer models by inducing cell cycle arrest and apoptosis (231, 232). Unfortunately, these effects could not be translated into clinical use as a kinase inhibitor mono-therapy, due to limited efficacy and a myriad of toxicities (233). Currently, combination trials of seliciclib with sapacitabine in BRCA-mutant solid tumors are ongoing.

Dinaciclib (SCH 727965) inhibits CDKs 1, 2, 5, and 9 with a similar potency (IC_50_ = 3, 1, 1, and 4 nM, respectively), and displays better selectivity for CDKs relative to other protein kinases (234). The compound induces cell cycle arrest and apoptosis in multiple cancer cell lines representing a broad range of cancer types and showed *in vivo* antitumor efficacy after intraperitoneal administration in tumor xenograft models (130, 136, 141, 151, 162, 208). Dinaciclib was well tolerated in phase I clinical trials when administered weekly for 3 weeks, leading to its progression into phase II trials against solid cancers (235). In subsequent breast and lung cancer trials, dinaciclib did not perform better than comparator agents, resulting in premature termination (236, 237). Encouraged by the positive outcome of flavopiridol in CLL patients (199), dinaciclib was investigated in relapsed and refractory CLL and 54% of patients showed a partial response with limited side-effects (e.g. cytopenia and tumor lysis syndrome) (137). Hence, dinaciclib progressed to a phase III trial with the anti-CD20 monoclonal antibody ofatumumab as comparator (200). Although dinaciclib did demonstrate efficacy and was tolerated, the phase III trial was terminated early (due to reasons unrelated to safety and efficacy), precluding definitive conclusions (200). Besides CLL, dinaciclib has also been trialed as a single agent in patients with relapsed multiple myeloma having a partial response rate of 11% (238). Currently, clinical trials are investigating the efficacy of dinaciclib in combination with a BCL-2 inhibitor (venetoclax) for R/R AML (NCT03484520), immunotherapy (pembrolizumab) for R/R hematological malignancies (NCT02684617), and a Poly (ADP-ribose) polymerase (PARP) inhibitor (veliparib) against solid cancers (NCT01434316).

Over the years, numerous other small molecule CDK9 inhibitors have been discovered (e.g., CDKI-73, TG02) (198, 239). These compounds are currently either in preclinical development or in the early stages of clinical trials. **Supplementary Table 1** provides detailed information on the development stages of various CDK9 inhibitors.

### Second Generation CDK9 Inhibitors

BAY1143572 (atuveciclib), a benzyl sulfoximine, is one of the most selective and potent CDK9 inhibitors (IC_50_ = 6 nM) currently being evaluated in clinical trials (192). The compound inhibited the proliferation of cancer cell lines at sub-micromolar concentrations and suppressed the growth of subcutaneous xenograft models of AML (134, 192), TNBC (157), lymphoma (143, 146), and esophageal (165) cancer. BAY1143572 inhibited the phosphorylation of RNAP II CTD on Ser2, downregulated MCL-1 and MYC, and induced apoptosis. Based on these findings, BAY1143572 is being evaluated in two phase I clinical trials involving patients with advanced cancers (gastric cancer, TNBC, and DLBCL; NCT01938638) and acute leukemias (NCT02345382), with results yet to be reported. In a follow-up lead optimization, a related compound, BAY1251152, was identified with increased potency (IC_50_ for CDK9 = 3 nM) and solubility to allow intravenous (IV) administration (240). BAY1251152 demonstrated antitumor efficacy in AML xenograft models and progressed to phase I clinical evaluation (240). BAY1251152 was administered once weekly for 3 weeks as a 30 minute IV infusion to patients with metastatic solid cancers or aggressive non-Hodgkin lymphoma and caused the disease to stabilize in 12 out of 31 patients with a manageable safety profile (NCT02635672) (241). This compound is also currently under investigation in patients with advanced hematological malignancies (phase I, NCT02745743).

AZD4573 is a potent inhibitor of CDK9 (IC_50_ < 4 nM) having more than ten-fold selectivity for CDK9 over CDKs 1 – 7 (242). It downregulated MCL-1 and induced rapid apoptosis in a large panel of hematologic cancer cell lines after a short exposure (*i.e.*, 6 h) (242). The compound led to the regression of subcutaneous tumor xenografts and disseminated models of AML after twice weekly dosing through the intraperitoneal route alone as single agent or in combination with venetoclax (242). Consequently, AZD4573 is currently being evaluated in a phase I clinical trial for patients with hematological malignancies (NCT03263637).

i-CDK9 is a compound of pico-molar potency with a 600-fold selectivity for CDK9 over other CDKs (243). Besides CDKs, i-CDK9 inhibits dual-specificity tyrosine-phosphorylation-regulated kinases (DYRK) 1A and 1B, although at a lower potency compared to CDK9. i-CDK9 reduced the phosphorylation of CTD on Ser2 and SPT5 of DSIF on Thr775, downregulated MCL-1, and induced apoptosis in HeLa cells. Consistent with CDK9 inhibition, chromatin immunoprecipitation followed by parallel DNA sequencing (ChIP-seq) indicated that greater than 50% of genes in HeLa cells contained RNAP II paused in their promoter-proximal regions following incubation with i-CDK9 (243). Of interest was a small group of genes including MYC, which displayed a rebound expression in the presence of i-CDK9 prior to complete suppression of CTD Ser2 phosphorylation. This paradox was ascribed to a compensatory release of CDK9 from 7SK snRNP by BRD4 (**Figure 5**), which was abrogated by a combined inhibition of CDK9 and BRD4 (using JQ1). Due to its poor pharmacological properties, i-CDK9 has not progressed into clinical testing.

NVP-2 is an aminopyrimidine based inhibitor and a chemical analogue of i-CDK9 which potently and selectively inhibits CDK9 (IC_50_ = 0.5 nM) (244). NVP-2 displayed antiproliferative activity against numerous leukemia cell lines, associated with downregulation of MCL-1 and induction of apoptosis. RNA sequencing and ChIP-seq analysis have shown that NVP-2 downregulated a large percentage of total mRNA in MOLT4 cells and increased the localization of RNAP II near promoter-proximal regions (244). Concurrently, the same research team also described a novel method of achieving selectivity by linking SNS032 (a non-selective CDK9 inhibitor) to a thalidomide (THAL) moiety that recruits E3 ligase cereblon (CRBN) to catalyze the proteasomal degradation of CDK9. Through such a method, the specific degradation of CDK9 by THAL-SNS-032 was achieved, which resulted in prolonged antiproliferative activity and apoptosis compared to ATP-competitive inhibition (e.g., NVP-2, SNS032). The pharmacokinetic and pharmacodynamic characteristics of these compounds have not been reported yet.

## Discussion

In summary, CDK9 along with cyclin T1 (constituting the P-TEFb complex) plays a key role in transcription by allowing RNAPII to facilitate the productive elongation of transcripts. Its roles extend beyond transcriptional elongation with functions in the cell cycle, differentiation, DNA repair, and transcriptional initiation and termination. Detailed structural characterization has revealed a conserved cyclin-dependent phosphor-transfer mechanism across CDKs, with some subtle differences in substrate recognition and cyclin binding for CDK9. P-TEFb activity is regulated by sequestering into an inactive complex and various post-translational modifications. Given its pivotal functions, when CDK9 becomes overactive in many hematological and solid cancers there is a continuous production of short-lived proteins that maintain the survival of cancer cells. This addiction to transcription makes cancer cells highly susceptible to the inhibition of CDK9 relative to non-transformed cells. Understanding the biology and function of CDK9 has advanced dramatically since its discovery in 1994 (13) and this has had a positive impact on the design and the use of specific inhibitors as a potential strategy for the treatment of several diseases. In line with this, several first generation CDK9 inhibitors have been developed and tested in clinical trials mostly in combination with conventional chemotherapeutic agents. Unfortunately, these investigational new drug entities have been hampered by severe adverse effects and to date none of them have made it to clinical approval (**Supplementary Table 1**).

Nevertheless, the authors predict that future development will be guided by insights into the molecular structure and function of CDK9, which will serve as the driving force for further improvements in the potency and specificity of novel inhibitors. Meanwhile, a more advanced understanding of its biology is likely to pave the way for establishing a sounder basis for the future value of CDK9 inhibitors for cancer therapy. One missing piece of knowledge in the CDK9 puzzle is a full validation of this target for cancer treatment. Experimental *in vivo* validation would be best assessed with CDK9-deficient mice. Unfortunately, knockout of CDK9 or its binding partner cyclin T2, is embryonically fatal to the mouse (245, 246). Designs for future studies might use conditional genetic knockout of the CDK9 or cyclin T1/2 genes in various established cancer models to provide more information about the role of the P-TEFb complex in tumor formation. The use of specific inhibitors of CDK9 as chemical probes may well be applied to finally confirm the outcomes from these sophisticated models. To this end, new hope has arisen in recent years from second generation inhibitors, with their much-improved specificity for CDK9 inhibition (**Supplementary Table 1**). Overall, a holistic understanding of the underlying CDK9 biology will be a prerequisite to optimizing the use of novel kinase inhibitors as mono and/or adjuvant therapies for the future treatment of various neoplastic disorders.

## Author Contributions

AA: wrote the manuscript. HA, RM, and SW: conceptualized and critically revised the manuscript. All authors contributed to the article and approved the submitted version.

## Conflict of Interest

The authors declare that the research was conducted in the absence of any commercial or financial relationships that could be construed as a potential conflict of interest.
