# Supplementary material for: CDK9: A Comprehensive Review of Its Biology, and Its Role as a Potential Target for Anti-Cancer Agents
Source: Front Oncol. 2021 May 10;11:678559. doi: 10.3389/fonc.2021.678559 (PMC8143439; doi:10.3389/fonc.2021.678559)
Supplement: Supplementary file 1 [file Table_1.docx]

**Supplementary Table 1:** CDK9 inhibitors in different stages of development

| **Compounds** | **Kinase Activity**  **(IC_50,_ µM)** | **Cellular Activity**  **(GI_50_, µM)*** | **Preclinical Studies**  **Mode of Action** | **Stage of Development**** | **Ref** |
| --- | --- | --- | --- | --- | --- |
| 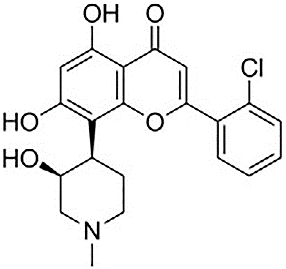**Flavopiridol** | **CDK9T1 (0.007)**  CDK1B (0.050)  CDK2A (0.070)  CDK4D1 (0.100)  CDK6D (0.395)  CDK7H (0.200)  CDK8C (0.020) | Prostate (PC3, 0.010)  Colon (HCT116, 0.013)  Ovarian (A2780, 0.015)  Pancreatic (Mia PaCa-2, 0.036) | G_1_/S, G_2_/M arrest; Apoptosis:   - Inhibits the phosphorylation of Rb and RNAP II on Ser2, - Downregulates MCL-1, BCL-2, and XIAP, - Increases caspases 3/8 activity, cleavage of PARP, and P53 activity. | **Phase II** in different hematological and solid cancers  **Phase I**: Ongoing   - R/R or newly diagnosed AML in combination with venetoclax, cytarabine, cytarabine/daunorubicin - In combination with decitabine in myelodysplastic syndrome | ^(1-4)^ |
| **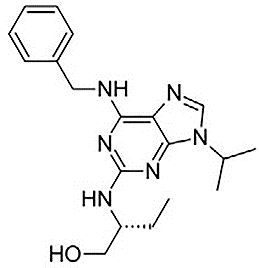Roscovitine** | **CDK9T1 (0.950)**  CDK1B (2.690)  CDK2A (0.100)  CDK4/6D (>10.0)  CDK5P35 (0.160)  CDK7H (0.490) | Lung (21.3)  Colon (13.2)  Renal (12.6)  Breast (14.2)  Uterine (9.9) | G_1_/S, G_2_/M arrest; Apoptosis:   - Accumulation of P53 and cleavage of PARP, - Decreases the expression of XIAP. | **Phase I**:   - BRCA mutant advanced solid tumour in combination with Sapacitabine   **Phase II**: Ongoing   - Cushing disorder and cystic fibrosis | ^(5, 6)^ |
| **Dinaciclib**  **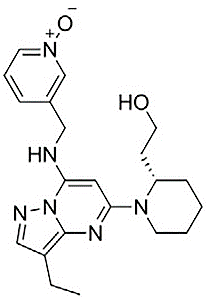** | **CDK9 (0.004)**  CDK1 (0.003)  CDK2 (0.001)  CDK5 (0.001) | Leukemia (0.006)  Lung, SCLC (0.006)  Lymphoma (0.007)  Breast (0.008)  Liver (0.008)  Melanoma (0.009)  Bladder (0.010)  Prostate (0.012)  Ovarian (0.014)  Pancreatic (0.015)  Colon (0.017) | G_1_/S, G_2_/M arrest; Apoptosis:   - Inhibits the phosphorylation of Rb and RNAP II on Ser2, - Downregulates MCL-1, activates caspase activity, and increases cleavage of PARP. | **Phase I – III** trials in CLL, breast cancer, myeloma, pancreatic cancer (either alone or in combination)  **Phase I**: Ongoing: in combination with   - venetoclax in R/R AML - pembrolizumab in R/R hematological malignancies - veliparib in solid cancers | ^(7)^ |
| **SNS 032**  **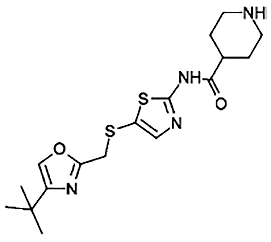** | **CDK9T (0.004)**  CDK1B (0.480)  CDK2A (0.038)  CDK4D (0.925)  CDK6D (>1.0)  CDK7H (0.062) | Ovarian (A2780, 0.039)  Lung (A549, 0.043)  Colon (HCT116, 0.070)  Breast (MCF-7, 0.184; MDA-MB-435, 0.133) | G_1_/S, G_2_/M arrest; Apoptosis:   - Inhibits the phosphorylation of Rb and RNAP II on Ser 2 and 5, - Downregulates MCL-1 and XIAP. | **Phase I** trials in CLL and advanced solid tumors | ^(8-10)^ |
| **TG02**  **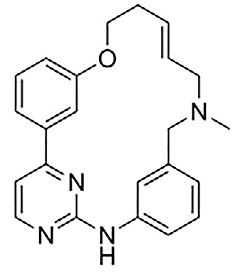** | **CDK9 (0.003)**  CDK1 (0.009)  CDK2 (0.005)  CDK4 (>100.0)  CDK6 (0.113)  CDK5 (0.004)  CDK7 (0.037) | Leukemia (0.130)  Melanoma (0.329)  Lung (0.250)  Colon (0.305)  Normal fibroblast (0.353) | G_1/_S arrest; Apoptosis:   - Inhibits the phosphorylation of Rb and RNAP II on Ser 2, - Decreases the expression of MCL-1 and increases cleavage of PARP. | **Phase I** trials in hematological malignancies   - CLL, small lymphocytic lymphoma, AML, ALL   **Phase I/II**: Ongoing   - High-grade gliomas - Alone or in combination with radiotherapy or Temozolomide in Anaplastic Astrocytoma or Glioblastoma | ^(11)^ |
| 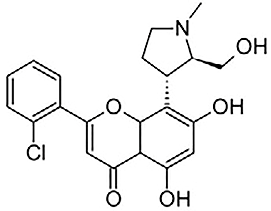**P276-00** | **CDK9T1 (0.020)**  CDK1B (0.079)  CDK2E (2.540)  CDK4D1 (0.063)  CDK6D3 (0.396)  CDK7H (2.870) | Bladder (T-24, 0.390)  Sarcoma (U2OS, 0.400)  Cervical (SiHa, 0.420)  Breast (MCF-7, 0.520)  Prostate (PC3, 0.56)  Colon (0.594)  Leukemia (HL60, 0.750)  Lung (H460, 0.800) | G_1_/S arrest; Apoptosis:   - Downregulates cyclin D1 and CDK4, - Inhibits the phosphorylation of Rb and RNAP II on Ser2, - Downregulates MCL-1, activates caspase 3 activity, and induces cleavage of PARP. | **Phase I/II** trials in pancreatic, head and neck cancers, melanoma, and MM   - As a single agent or in combination with gemcitabine or radiation therapy | ^(12, 13)^ |
| **AT7519**  **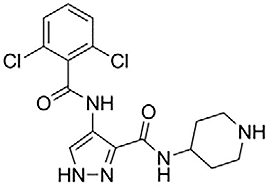** | **CDK9T1 (<0.010)**  CDK1B (0.210)  CDK2A (0.047)  CDK4D1 (0.100)  CDK6D3 (0.170)  CDK5P35 (0.013)  CDK7H (2.400) | Leukemia (0.249)  Breast (0.260)  Ovarian (0.375)  Lung (0.390)  Colon (0.397)  Lymphoma (0.517)  Uterine (MESSA, 0.660) | G_1_/S, G_2_/M arrest; Apoptosis:   - Inhibits the phosphorylation Rb and RNAP II on Ser 2 and 5, - Downregulates MCL-1 and XIAP. | **Phase I/II** trials in multiple myeloma, mantle cell lymphoma, refractory NHL, and refractory CLL  **Phase I**: Ongoing   - In combination with onalespib in advanced solid cancers | ^(14)^ |
| **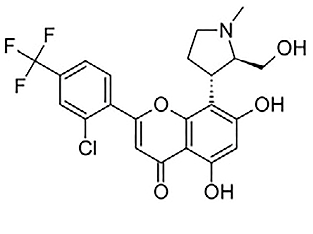Voruciclib** | **CDK9T1 (0.002)**  CDK1B (0.005)  CDK4D1 (0.004)  CDK6D1 (0.003) | Range against thirty-human cancer cell lines (0.500 – 3.900) | G_1_/S, G_2_/M arrest; Apoptosis:   - Inhibits the phosphorylation of Rb and RNAP II on Ser 2, - Downregulates cyclinD1, MCL-1, and BCL-2, - Induces P53 and cleavage of PARP. | **Phase I** trials in advanced refractory malignancies  **Phase I:** Ongoing   - B-cell malignancies and AML | ^(15, 16)^ |
| **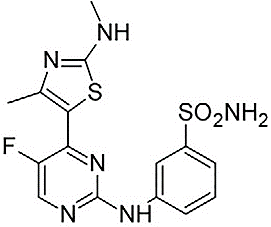CDKI-73** | **CDK9 (*K_i_* = 0.004)**  CDK1 (0.004)  CDK2 (0.003)  CDK4 (0.009)  CDK6 (0.052)  CDK7 (0.091) | Ovarian (0.007)  Cervical (0.031)  Colon (0.044)  Prostate (0.050)  Epithelial (0.057)  Breast (0.065)  Leukemia (0.167)  Pancreatic (0.573)  Normal B cells (40.0) | Apoptosis:   - Inhibits the phosphorylation of RNAP II on Ser2, - Activates caspase 3/7 activity and increases cleavage of PARP, - Inhibits the expressions of MCL-1, XIAP, BCL-2, and MYC. | Phase I trials in advanced AML | ^(17, 18)^ |
|  |  |  |  |  |  |
| **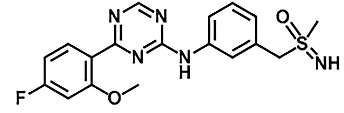BAY1143572** | **CDK9T1 (0.006)**  CDK1B (1.1)  CDK2E (1.0)  CDK3E (0.890)  CDK5P35 (1.600)  CDK6D3 (>10.0)  CDK7H (>10.0) | Leukemia (0.385)  NK-cell leukemia/lymphoma (0.387)  Adult T-cell leukemia (0.716)  TNBC (2.298)  Esophageal (1.358) | Apoptosis:   - Inhibits the phosphorylation of RNAP II on Ser2, - Downregulates MYC, MCL-1, and induces cleavage of PARP. | **Phase I**:   - Advanced cancer (gastric cancer, TNBC, and DLBCL) - Acute leukemias | ^(19-23)^ |
| **BAY1251152**  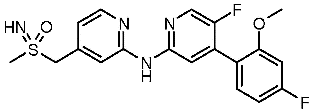 | **CDK9T1 (0.003)**  CDK2E (0.360) | Cervical (HeLa, 0.11; HeLa-MaTu-ADR, 0.033)  Lung (NCI-H460, 0.075)  Prostate (DU145, 0.033)  Colon (Caco_2_, 0.062)  Melanoma (B16F10 (mouse), 0.24)  Ovarian (A2780, 0.11)  Leukemia (MOLM-13, 0.029) | - Demonstrated antitumor efficacy in AML xenograft models. | **Phase I**:   - Advanced or metastatic solid tumors or aggressive and refractory NHL - Advanced hematological malignancies | ^(24, 25)^ |
| **AZD4573**  **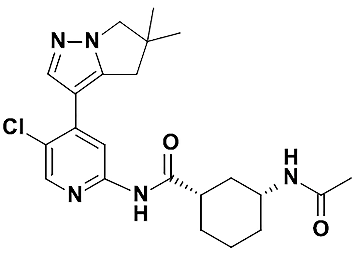** | **CDK9T1 (<0.004)**  Greater than 10-fold selectivity over  CDKs 1, 2, and 6.  Greater than 100-fold selectivity over CDKs 4, 5, 7, and 12 | Hematologic cancer cell lines (0.025)  Solid cancer cell lines (0.774) | Apoptosis:   - Inhibits the phosphorylation of RNAP II on Ser2, - Downregulates MCL-1 and activates caspase 3. | **Phase I**:   - Relapsed and refractory hematological malignancies | ^(26)^ |
| **i-CDK9**  **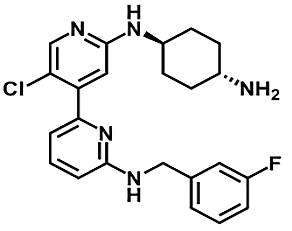** | **CDK9T1 (≤ 0.0004)**  CDK1B (1.700)  CDK2A (0.240)  CDK4D1 (1.800)  CDK7H (2.0)  CDK8C (>1.0) |  | - Inhibits the phosphorylation of RNAP II on Ser2 and SPT5 of DSIF, - Downregulates MCL-1 and induces cleavage of PARP. | Preclinical | ^(2)^ |
| **NVP-2**  **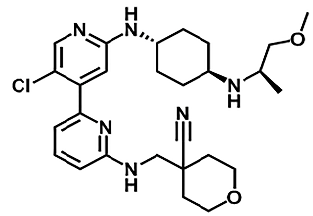** | **CDK9T1 (< 0.0005)**  CDK1B (0.584)  CDK2A (0.706)  CDK5P25 (1.050)  CDK7H (>10.0)  CDK8C (>10.0) | Leukemia (0.013) | Apoptosis:   - Inhibits the phosphorylation of RNAP II on Ser2, 5, and 7, - Downregulates MCL-1 and induces cleavage of PARP and caspase 3. | Preclinical | ^(27)^ |

*GI_50_ represents average values unless cell lines are specified.

**From [www.clinicaltrial.gov](http://www.clinicaltrial.gov) (Accessed on 15/12/2020); CDKI-73 from www.aucentra.com.

**References**

1. Kim KS, Sack JS, Tokarski JS, Qian L, Chao ST, Leith L, et al. Thio- and oxoflavopiridols, cyclin-dependent kinase 1-selective inhibitors: synthesis and biological effects. *J Med Chem* (2000) 43(22):4126-34. doi: 10.1021/jm000231g.

2. Lu H, Xue Y, Yu GK, Arias C, Lin J, Fong S, et al. Compensatory induction of MYC expression by sustained CDK9 inhibition via a BRD4-dependent mechanism. *Elife* (2015) 4:e06535. doi: 10.7554/eLife.06535.

3. Chen R, Keating MJ, Gandhi V, Plunkett W. Transcription inhibition by flavopiridol: mechanism of chronic lymphocytic leukemia cell death. *Blood* (2005) 106(7):2513-9. doi: 10.1182/blood-2005-04-1678.

4. Whittaker SR, Mallinger A, Workman P, Clarke PA. Inhibitors of cyclin-dependent kinases as cancer therapeutics. *Pharmacol Ther* (2017) 173:83-105. doi: 10.1016/j.pharmthera.2017.02.008.

5. McClue SJ, Blake D, Clarke R, Cowan A, Cummings L, Fischer PM, et al. In vitro and in vivo antitumor properties of the cyclin dependent kinase inhibitor CYC202 (R-roscovitine). *Int J Cancer* (2002) 102(5):463-8. doi: 10.1002/ijc.10738.

6. Meijer L, Borgne A, Mulner O, Chong JP, Blow JJ, Inagaki N, et al. Biochemical and cellular effects of roscovitine, a potent and selective inhibitor of the cyclin-dependent kinases cdc2, cdk2 and cdk5. *Eur J Biochem* (1997) 243(1-2):527-36. doi: 10.1111/j.1432-1033.1997.t01-2-00527.x.

7. Parry D, Guzi T, Shanahan F, Davis N, Prabhavalkar D, Wiswell D, et al. Dinaciclib (SCH 727965), a novel and potent cyclin-dependent kinase inhibitor. *Mol Cancer Ther* (2010) 9(8):2344-53. doi: 10.1158/1535-7163.MCT-10-0324.

8. Conroy A, Stockett DE, Walker D, Arkin MR, Hoch U, Fox JA, et al. SNS-032 is a potent and selective CDK 2, 7 and 9 inhibitor that drives target modulation in patient samples. *Cancer Chemother Pharmacol* (2009) 64(4):723-32. doi: 10.1007/s00280-008-0921-5.

9. Chen R, Wierda WG, Chubb S, Hawtin RE, Fox JA, Keating MJ, et al. Mechanism of action of SNS-032, a novel cyclin-dependent kinase inhibitor, in chronic lymphocytic leukemia. *Blood* (2009) 113(19):4637-45. doi: 10.1182/blood-2008-12-190256.

10. Xie G, Tang H, Wu S, Chen J, Liu J, Liao C. The cyclin-dependent kinase inhibitor SNS-032 induces apoptosis in breast cancer cells via depletion of Mcl-1 and X-linked inhibitor of apoptosis protein and displays antitumor activity in vivo. *Int J Oncol* (2014) 45(2):804-12. doi: 10.3892/ijo.2014.2467.

11. Goh KC, Novotny-Diermayr V, Hart S, Ong LC, Loh YK, Cheong A, et al. TG02, a novel oral multi-kinase inhibitor of CDKs, JAK2 and FLT3 with potent anti-leukemic properties. *Leukemia* (2012) 26(2):236-43. doi: 10.1038/leu.2011.218.

12. Joshi KS, Rathos MJ, Joshi RD, Sivakumar M, Mascarenhas M, Kamble S, et al. In vitro antitumor properties of a novel cyclin-dependent kinase inhibitor, P276-00. *Mol Cancer Ther* (2007) 6(3):918-25. doi: 10.1158/1535-7163.MCT-06-0613.

13. Manohar SM, Rathos MJ, Sonawane V, Rao SV, Joshi KS. Cyclin-dependent kinase inhibitor, P276-00 induces apoptosis in multiple myeloma cells by inhibition of Cdk9-T1 and RNA polymerase II-dependent transcription. *Leuk Res* (2011) 35(6):821-30. doi: 10.1016/j.leukres.2010.12.010.

14. Squires MS, Feltell RE, Wallis NG, Lewis EJ, Smith DM, Cross DM, et al. Biological characterization of AT7519, a small-molecule inhibitor of cyclin-dependent kinases, in human tumor cell lines. *Mol Cancer Ther* (2009) 8(2):324-32. doi: 10.1158/1535-7163.MCT-08-0890.

15. Joshi KS, Padgaonkar A, Rathos M, Wagh V, Manohar S, Bhatia D, et al. P1446A-05: a new oral cyclin-dependent kinase inhibitor with potent preclinical antitumor activity. *Cancer Research* (2012) 72. doi: 10.1158/1538-7445.Am2012-3054.

16. Dey J, Deckwerth TL, Kerwin WS, Casalini JR, Merrell AJ, Grenley MO, et al. Voruciclib, a clinical stage oral CDK9 inhibitor, represses MCL-1 and sensitizes high-risk Diffuse Large B-cell Lymphoma to BCL2 inhibition. *Sci Rep* (2017) 7(1):18007. doi: 10.1038/s41598-017-18368-w.

17. Walsby E, Pratt G, Shao H, Abbas AY, Fischer PM, Bradshaw TD, et al. A novel Cdk9 inhibitor preferentially targets tumor cells and synergizes with fludarabine. *Oncotarget* (2014) 5(2):375-85. doi: 10.18632/oncotarget.1568.

18. Lam F, Abbas AY, Shao H, Teo T, Adams J, Li P, et al. Targeting RNA transcription and translation in ovarian cancer cells with pharmacological inhibitor CDKI-73. *Oncotarget* (2014) 5(17):7691-704. doi: 10.18632/oncotarget.2296.

19. Lucking U, Scholz A, Lienau P, Siemeister G, Kosemund D, Bohlmann R, et al. Identification of Atuveciclib (BAY 1143572), the First Highly Selective, Clinical PTEFb/CDK9 Inhibitor for the Treatment of Cancer. *ChemMedChem* (2017) 12(21):1776-93. doi: 10.1002/cmdc.201700447.

20. Scholz A, Oellerich T, Hussain A, Lindner S, Luecking U, Walter AO, et al. BAY 1143572, a first-in-class, highly selective, potent and orally available inhibitor of PTEFb/CDK9 currently in Phase I, shows convincing anti-tumor activity in preclinical models of acute myeloid leukemia (AML). *Cancer Research* (2016) 76. doi: 10.1158/1538-7445.Am2016-3022.

21. Kinoshita S, Ishida T, Ito A, Narita T, Masaki A, Suzuki S, et al. Cyclin-dependent kinase 9 as a potential specific molecular target in NK-cell leukemia/lymphoma. *Haematologica* (2018) 103(12):2059-68. doi: 10.3324/haematol.2018.191395.

22. Narita T, Ishida T, Ito A, Masaki A, Kinoshita S, Suzuki S, et al. Cyclin-dependent kinase 9 is a novel specific molecular target in adult T-cell leukemia/lymphoma. *Blood* (2017) 130(9):1114-24. doi: 10.1182/blood-2016-09-741983.

23. Tong z, Mejia A, Veeranki O, Verma A, Correa A, Patel V, et al. Abstract 3859: Targeting CDK9 and MCL-1 by a New CDK9/p-TEFB Inhibitor with and without 5-fluorouracil in esophageal adenocarcinoma. *Cancer Research* (2019) 79(13):3859-. doi: 10.1158/1538-7445.am2019-3859.

24. Luecking UT, Scholz A, Kosemund D, Bohlmann R, Briem H, Lienau P, et al. Identification of potent and highly selective PTEFb inhibitor BAY 1251152 for the treatment of cancer: from p.o. to i.v. application via scaffold hops. *Cancer Research* (2017) 77. doi: 10.1158/1538-7445.Am2017-984.

25. Diamond JR, Moreno V, Lim EA, Cordoba R, Cai C, Ince SJ. Phase I dose escalation study of the first-in-class selective PTEFb inhibitor BAY 1251152 in patients with advanced cancer: Novel target validation and early evidence of clinical activity. *Journal of Clinical Oncology* (2018) 36(15). doi: DOI 10.1200/JCO.2018.36.15_suppl.2507.

26. Cidado J, Boiko S, Proia T, Ferguson D, Criscione SW, San Martin M, et al. AZD4573 Is a Highly Selective CDK9 Inhibitor That Suppresses MCL-1 and Induces Apoptosis in Hematologic Cancer Cells. *Clin Cancer Res* (2020) 26(4):922-34. Epub 2019/11/09. doi: 10.1158/1078-0432.CCR-19-1853.

27. Olson CM, Jiang B, Erb MA, Liang Y, Doctor ZM, Zhang Z, et al. Pharmacological perturbation of CDK9 using selective CDK9 inhibition or degradation. *Nat Chem Biol* (2018) 14(2):163-70. doi: 10.1038/nchembio.2538.
